# Modification of aggregation-prone regions of Arabidopsis glutamyl-tRNA reductase leads to increased stability while maintaining enzyme activity

**DOI:** 10.3389/fpls.2025.1556843

**Published:** 2025-03-13

**Authors:** Shuiling Ji, Peng Wang, Bernhard Grimm

**Affiliations:** ^1^ Key Laboratory of Pesticide & Chemical Biology of Ministry of Education, Hubei Key Laboratory of Genetic Regulation and Integrative Biology, School of Life Sciences, Central China Normal University, Wuhan, China; ^2^ Institute of Biology/Plant Physiology, Humboldt-Universität zu Berlin, Berlin, Germany; ^3^ School of Biological Sciences, The University of Hong Kong, Hong Kong, Hong Kong SAR, China; ^4^ State Key Laboratory of Agrobiotechnology, The Chinese University of Hong Kong, Hong Kong, Hong Kong SAR, China

**Keywords:** GluTR, aggregation-prone region, cpSRP43, chaperone, ALA synthesis, post-translational control

## Abstract

The aggregation-prone region (APR) is a hydrophobic polypeptide motif that promotes protein aggregation, most commonly in the unfolded or misfolded state. It has been described that chaperones can shield the APRs of proteins, thereby preventing aggregate formation during *de novo* protein synthesis and stress response. Glutamyl-tRNA reductase (GluTR) is a key enzyme in tetrapyrrole biosynthesis (TBS) which catalyzes the rate-limiting step of 5-aminolevulinic acid synthesis. The GluTR sequence contains two APRs located at the N-terminus, which are suggested to be associated with the dysregulation of protein homeostasis during folding and refolding processes or under stress conditions. It remains open if these APRs directly contribute to GluTR aggregation *in vivo*, and how their removal or the modification might impact the aggregation and stability. In this study, we altered and removed the GluTR-APRs to investigate their effects on the stability and enzymatic activity of GluTR. Deletion of the APRs has been shown to be highly disruptive to the structure of GluTR, and a substitution mutation of V→P in each APR has also lowered the GluTR stability and activity. In contrast, the mutation V→T resulted in a modest reduction (18–30%) in GluTR aggregation *in vitro*, which was associated with a 27% improvement in GluTR stability *in vivo* relative to the wild-type enzyme. These results indicate that a point mutation in APR can improve GluTR stability without significantly affecting enzyme activity, thus imposing a potential direction for bioengineering of GluTR to improve productivity of the TBS pathway in plants.

## 1 Introduction

Tetrapyrrole biosynthesis (TBS) is one of the most essential biochemical pathways in living organisms, producing end-products such as chlorophyll (Chl), heme, bilins (including phytochromes), and siroheme. These compounds play essential roles in various biological processes. For instance, Chl is vital for photosynthesis, where it absorbs, transmits, and converts sunlight into chemical energy, initiating charge separation at the photoreaction center of the photosynthetic system (Wang and Grimm, 2021). Heme acts as a cofactor in many enzymes for electron transfer, redox reaction, scavenging of reactive oxygen species and binding of gases and is proposed to play a potential role in retrograde signaling from plastids (Wang et al., 2022). Phytochromobilin serves as the chromophore for phytochromes during photomorphogenesis (Wang et al., 2022).

In plants, glutamyl-tRNA reductase (GluTR) facilitates the reduction of tRNA-conjugated glutamate to glutamate-1-semialdehyde (GSA), which is then converted to 5-aminolevulinic acid (ALA) by GSA aminotransferase (GSAT) (Ilag and Jahn, 1992; Smith et al., 1992; Richter et al., 2019). The production of ALA represents the rate-limiting step in the TBS pathway and is controlled by the precise regulation of the stability and activity of GluTR (Apitz et al., 2014, 2016; Wang et al., 2018). Eight ALA molecules are converted into protoporphyrin IX (Proto IX) through a series of six enzymatic reactions. Then, Proto IX is routed either through the iron-dependent pathway for heme synthesis or the magnesium-dependent pathway for Chl production (Tanaka and Tanaka, 2007; Richter et al., 2019).

A recent review highlighted three key metabolic checkpoints in the post-translational regulation of TBS: GluTR, Magnesium chelatase (MgCh), and protochlorophyllide oxidoreductase (POR) (Wang et al., 2022). GluTR, as the rate-limiting enzyme in TBS, plays a critical role in controlling ALA availability during photoperiodic growth by regulating its activity, stability, and distribution (Wang et al., 2022). Several interacting factors have been identified that modulate GluTR activity and stability, underscoring the importance of post-translational regulation (Richter et al., 2019). Notable modifications include (i) redox regulation by NTRC (NADPH-dependent TRX reductase C) and TRX (thioredoxin) isoforms (Richter et al., 2013; Pérez-Ruiz et al., 2017; Wittmann et al., 2021), (ii) dark- and low-light-dependent inhibition through interactions with FLUORESCENCE IN BLUE LIGHT (FLU) (Meskauskiene et al., 2001; Hou et al., 2019), (iii) proteolysis by the plastid-localized Clp protease (Apitz et al., 2016), (iv) stabilization by the heme-binding GluTR-binding protein (GBP) (Czarnecki et al., 2011), whereby the GluTR interaction of GBP is attenuated by its competitive binding to heme (Richter et al., 2019), and last, but likely not least (v) thermal protection by the chaperone chloroplast signal-recognition particle 43 (cpSRP43) (Wang et al., 2018; Ji et al., 2021).

Proper protein folding is essential for functional activity. Plastid-localized proteins, such as GluTR, are initially synthesized as linear polypeptide chains during translation. These chains are imported into plastids as unfolded protein through the translocon complexes in the outer and inner envelope membranes of chloroplasts (TOC and TIC complexes) and finally refolded inside the plastids into their three-dimensional structures to perform their functions. However, proteins are prone to misfolding and incorrect folding, both can form aggregates caused by noncovalent interactions, including hydrophobic forces, hydrogen bonds, and van der Waals interactions (Sahin et al., 2011; Roberts, 2014; O’Brien et al., 2016). Several pathways or mechanisms of aggregation are outlined by (Meric et al., 2017). Among these, the hydrophobic polypeptide motifs, known as aggregation-prone regions (APRs), are pivotal in driving protein aggregation. Typically, APRs consist of 5–15 consecutive hydrophobic amino acids with a low net charge, and aggregation occurs when they are exposed, leading to self-association and the formation of intermolecular β-structured assemblies (De Baets et al., 2014). In properly folded proteins, APRs are usually buried in the hydrophobic core or at protein-protein interaction sites, where they are protected from the action of solvents (Beerten et al., 2012). Chaperones play a pivotal role in managing protein aggregation by recognizing exposed APRs and transiently binding to prevent aggregation. APR-mediated protein aggregation can disrupt the highly specific self-association process, if protein folding is not supported by multiple non-covalent interactions of chaperones that ensure the concealment of these APRs within the folded protein. The formation of stable complexes between chaperones and aggregation-prone proteins prevents the aggregation and insolubilization of the client proteins and keeps them in a soluble and functional state.

Since APRs promote protein aggregation, elimination or masking of these regions offers a potential strategy for engineering to make more stable and functional proteins. In contrast to globular proteins, naturally unfolding proteins are designed to avoid aggregation by reducing their hydrophobic content and increasing their net charge (Eichner and Radford, 2011). For instance, disrupting continuous sequences of hydrophobic amino acid residues by introducing charged side chains that function as gatekeepers can inhibit aggregation (Otzen et al., 2000). These gatekeepers prevent aggregation by (i) charge repulsion (R, K, E, D), (ii) large and flexible structures (R, K), or (iii) incompatibility with β-sheet formation (P, G). Supercharged proteins exhibit exceptional resistance to aggregation and refold efficiently (Lawrence et al., 2007). Similarly, the aggregation of therapeutic antibodies has been mitigated by identifying and modifying APRs through computational and experimental methods, enhancing their stability and efficacy (Chennamsetty et al., 2009). Various algorithms have been developed to predict APRs (De Baets et al., 2014; Meric et al., 2017; Housmans et al., 2023). For example, the TANGO algorithm identifies β-APR based on their conformational states and aggregation potential and predicts APRs as sequences containing at least five amino acids with a β-aggregation occupancy rate greater than 5% (Meric et al., 2017).

Two APRs consisting of 10 amino acid residues (aa, APR1-aa95–aa104) and 8 amino acid residues (APR2-aa149–aa156) have been reported in two neighboring β-sheets at the N-terminus of the mature Arabidopsis GluTR (Wang et al., 2018). Despite overexpressing GluTR in *Nicotiana tabacum* or *Arabidopsis thaliana*, there was no corresponding increase in ALA synthesis, likely because excess GluTR forms oligomers or aggregates (Schmied et al., 2011). The solubility of a recombinant N-terminal of GluTR (GluTR-N) lacking these APRs is significantly enhanced *in vitro*, suggesting that the APRs contribute to the aggregation propensity of GluTR-N *in vitro* (Wang et al., 2018). Additionally, cpSRP43 has been shown to protect GluTR from aggregation by preserving its native structure during heat stress (Wang et al., 2018; Ji et al., 2021), likely by binding to and masking the exposed APRs to ensure proper folding. However, the role of APRs in GluTR aggregation *in vivo* remains unclear. Whether they are also the reason for GluTR aggregation still needs to be studied. In this study, we investigated for the first time on a TBS enzyme the effects of APR-induced aggregation in GluTR by deletion and substitution of amino acid residues within the APR domains.

## 2 Materials and methods

### 2.1 Plant materials and growth conditions

*Arabidopsis thaliana* seedlings were cultivated under a 16-hour light/8-hour dark cycle, either in soil or on Murashige and Skoog (MS) medium. The MS medium composition included 4.43 g L^-1^ Murashige and Skoog salts with vitamins, 0.5 g L^-1^ MES buffer, and 8 g L^-1^ agar, adjusted to pH 5.7 using NaOH. Plants were grown in the chamber at 22°C with a light intensity of 100 µmol photons m^-2^ s^-1^. To apply heat shock, 14–18-day old seedlings were transferred from standard growth conditions to continuous light at 42°C. Unless otherwise stated, growth conditions with 16-hour light/8-hour darkness, 100 µmol photons m^-2^ s^-1^ light intensity, and a temperature of 22°C prevailed in this study. Details of the Arabidopsis lines applied are provided in **Supplementary Table S1**.

### 2.2 Stable transformation of *Arabidopsis thaliana*

Stable transformants of Arabidopsis were produced using a modified “floral dip” technique (Clough and Bent, 1998). Briefly, the overnight-grown *Agrobacterium tumefaciens* cultures were harvested, resuspended in an inoculation solution containing 0.5% MS, 0.05% MES, 5% sucrose, 0.05% Silwet L-77 (pH 5.7), and adjusted to OD_600_ = 0.8. This suspension was applied to the tips of the just unopened inflorescences. The plants were kept in low light for 1–2 days, and the transformation process was repeated 3–4 times over 2–3 days. After 14 days, primary transformants were selected using a selection marker, such as BASTA spraying.

### 2.3 Generation of Arabidopsis transgenic lines

APR deletion mutants and amino acid substitution variants were generated by amplifying the *HEMA1* coding sequence from total Arabidopsis ecotype Col-0 cDNA using primer pairs summarized in **Supplementary Table S2**. APR deletions within *HEMA1* were introduced via overlap PCR, while point mutations were generated using primers designed for specific amino acid changes. The resulting fragments were initially cloned into the pJet1.2 vector (Thermo Scientific) and the correct sequences were confirmed by sequencing. Subsequently, the verified inserts were excised and ligated into the binary vector pJA1 (Apitz et al., 2014), ensuring that gene expression was governed by the native promoter. Heterozygous *hema1* mutant plants were transformed with each pJA1 derivative using the GV2260 strain (*Agrobacterium tumefaciens*). Transgenic individuals were selected based on their resistance to the herbicide BASTA.

### 2.4 Analysis of chlorophyll content

Rosette leaves were collected and weighed to determine the fresh weight (FW) of plants grown under standard conditions. Chlorophyll content was analyzed as previously described (Ji et al., 2023).

### 2.5 Determination of ALA synthesis rates

ALA synthesis rates were determined as previously described (Ji et al., 2021).

### 2.6 RNA extraction and qRT-PCR

Total RNA was isolated from Arabidopsis leaves frozen in liquid nitrogen using the citric-acid extraction protocol (Onate-Sanchez and Vicente-Carbajosa, 2008). cDNA synthesis and Quantitative PCR (qPCR) were performed as previously described (Ji et al., 2023). The primers used for qRT-PCR are provided in **Supplementary Table S2**.

### 2.7 Protein extraction and immunoblot analysis

Protein extraction and immunoblot analysis was performed as described previously (Ji et al., 2021). Briefly, rosette seedlings (14–18 days old) from 3–6 individual plants were harvested. For plants grown under standard conditions, leaves were ground in liquid nitrogen, and total proteins were extracted using 2× Laemmli buffer, followed by incubation at 95°C for 10 minutes. Protein concentrations were measured and normalized to leaf fresh weight.

In heat shock experiments, rosette leaves were harvested before or after heat treatment, ground in liquid nitrogen, and proteins were extracted using PEB buffer. Protein concentrations were determined using the Pierce BCA Protein Assay Kit, and samples were adjusted to equal protein concentrations, supplemented with DTT, and incubated at 70°C for 20 minutes. Proteins were separated by SDS-PAGE, transferred to nitrocellulose membranes, and probed with specific antibodies. Immunoblot signals were detected using Clarity™ Western ECL reagents and a CCD camera.

### 2.8 Expression and purification of recombinant proteins

The expression and purification protocols for His-GluTR and His-GluTR(V99T/V151T) were previously detailed by (Ji et al., 2021). To construct a vector for expressing and purifying the recombinant GluTR(V99T/V151T), pQE80L containing the GluTR-encoding sequence without the transit peptide served as the template for full-length amplification. Primers containing the V→T mutations were designed according to (Laible and Boonrod, 2009). The specific primers utilized in this cloning process are listed in **Supplementary Table S2**.

### 2.9 Scattering assays of GluTR and its variant protein

To assess the effect of point mutations on protein aggregation, 2 µM His-GluTR and His-GluTR(V99T/V151T) in PBS buffer were subjected to a heat treatment at 42°C. The aggregation of GluTR induced by heat was monitored by measuring the turbidity through absorbance at 340 nm (A_340_) at two-minute intervals. These measurements were conducted using a temperature-controlled spectrophotometer (SPECTRA max M2; Molecular Devices) to ensure consistent and accurate detection of aggregation kinetics.

### 2.10 Isolation of intact chloroplasts

Isolation of intact chloroplasts was performed as described previously (Ji et al., 2021). Briefly, four-week-old *Arabidopsis thaliana* seedlings were homogenized in HB buffer, and filtered through Miracloth to remove debris. The filtrate was centrifuged at 500 × g for 8 minutes at 4°C, and the pellet was resuspended in RB buffer. Chloroplasts were isolated using a Percoll gradient, then washed with RB buffer for further purification. The chloroplast pellet was resuspended in lysis buffer containing inhibitor cocktail. Membrane proteins were extracted by solubilizing the chloroplasts with 0.2% (w/v) n-dodecyl-β-D-maltoside for 15 minutes. The isolated chloroplasts were either utilized immediately for downstream applications or stored by freezing in liquid nitrogen to preserve their integrity.

### 2.11 Blue Native PAGE and immunoblot analysis

Blue Native PAGE (BN-PAGE) was carried out following the protocol (Järvi et al., 2011). After protein separation, polyacrylamide lanes were excised and incubated with SDS sample buffer (50 mM Tris-HCl, pH 6.8, 2% w/v SDS, 10% glycerol, 0.002% w/v bromophenol blue, and 50 mM DTT) for one hour at room temperature and subsequently loaded onto 11% SDS-PAGE gels containing 6 M urea to dissociate the complexes. Following electrophoresis, proteins were transferred from the SDS-PA gels to nitrocellulose membranes, which were then incubated with specific antibodies. Immunoblotting signals were visualized using Clarity™ Western ECL reagents (Bio-Rad) and detected using a CCD camera (Intas Biopharmaceuticals), which is the ultimate approach to characterize protein complexes.

### 2.12 Image processing and data analysis

Image files, including Western blots, protein gels, and photographs, were processed using Adobe Photoshop CS3 and Inkscape (https://inkscape.org/). Protein blot signals were quantitatively analyzed through densitometry with ImageJ software (NIH). Graphical representations of the measurement results were generated using GraphPad Prism versions 8.0 or 9.1.2 (226) (www.graphpad.com). Statistical analyses were conducted utilizing Microsoft Excel 2016 or GraphPad Prism v9.1.2 (226).

## 3 Results

### 3.1 Generation and evaluation of APR deletion mutants

The structural domains of GluTR are illustrated in Figure 7A of a previous publication (Wang et al., 2018). The N-terminal catalytic region contains a regulatory domain (RED), to which GBP, the selector proteins ClpS and ClpF and the chaperone ClpC1 of Clp protease, and also cpSRP43 binds (Wang et al., 2018; Richter et al., 2019), followed by the catalytic domain, NADPH-binding domain, dimerization domain, and the C-terminal FLU-binding domain (FBD) (Wang et al., 2018). Computational analysis identified two APRs within the catalytic domain (Wang et al., 2018). To investigate the role of these APRs in GluTR function, we generated in an initial approach *HEMA1* genes with deletions for the APR regions and transformed *hema1* (*HEMA1* knockout) with the truncated *HEMA1* gene. The identified and selected mutants were designated as *HEMA1ΔAPR1*, *HEMA1ΔAPR2*, and *HEMA1ΔAPR1/2*, expressing three truncated versions of GluTR, named GluTRΔAPR1, GluTRΔAPR2, and GluTRΔAPR1/2, respectively.

**Figure 1:**
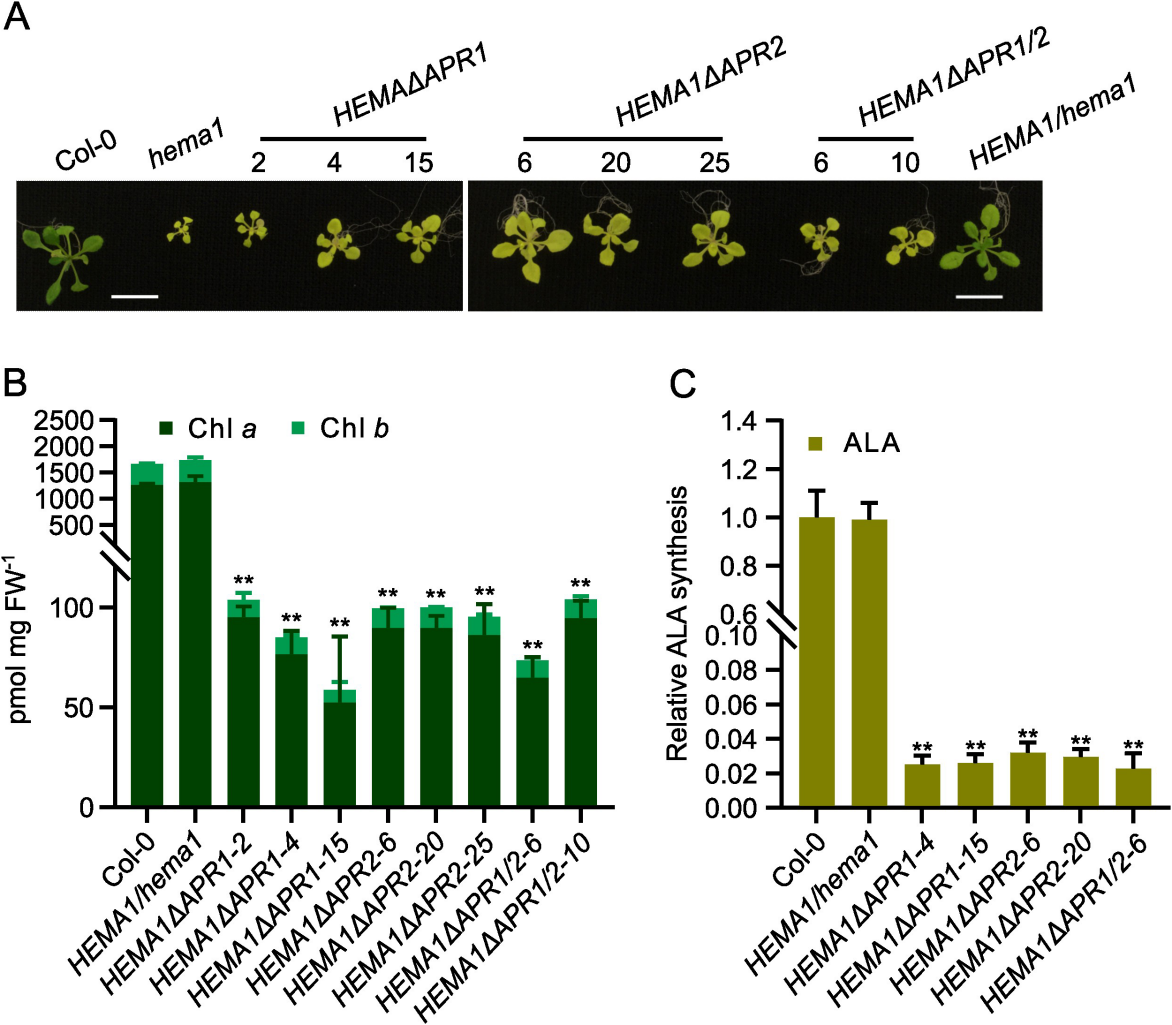
APR deletion mutants show severely impaired chlorophyll (Chl) biosynthesis. **(A)** Representative images of 16-day-old seedlings of Col-0, *hema1, HEMA1/hema1, HEMA1ΔAPR1, HEMA1ΔAPR2*, and *HEMA1ΔAPR1/2* grown under standard conditions (16 h light/8 h dark, 100 mmol photons m^-2^ s^-1^, 22°C). Scale bar: 1 cm. **(B, C)**, Chl content **(B)** and ALA synthesis rate **(C)** in 16-day-old seedlings grown under the same conditions as in **(A)**. FW: fresh weight. The relative ALA synthesis rates of the mutant lines were normalized to wild-type Col-0. All values are plotted as means ± s.d. (*n* = 3 independent biological repeats). Statistical analysis was performed using two-tailed Student’s t-tests. Asterisks indicate significant differences compared to Col-0: **P < 0.01.

Genotypic analysis of the primary transformants confirmed the successful integration of the T-DNA with the different truncated *HEMA1* gene sequences into homozygous *hema1* (**Supplementary Figure S1**). Phenotypic analysis of these mutants revealed yellowing leaves and stunted growth (**Figure 1A**). While the *HEMA1ΔAPR1* and *HEMA1ΔAPR1/2* lines grew only slightly faster than *hema1*, the growth of *HEMA1ΔAPR2* line reached the size of wild-type and complementing wildtype *HEMA1*/*hema1* lines. However, all transformed plants expressing *HEMA1* mutant genes were pale green similar to *hema1*. These findings were consistent with the reduced Chl content and diminished ALA synthesis rates in all transgenic *HEMA1ΔAPR* lines analyzed (**Figures 1B, C**), suggesting that the APRs deletion of GluTR severely impairs its function and abolishes Chl biosynthesis.

### 3.2 APR deletions compromise the stability and activity of GluTR

**Figure 2:**
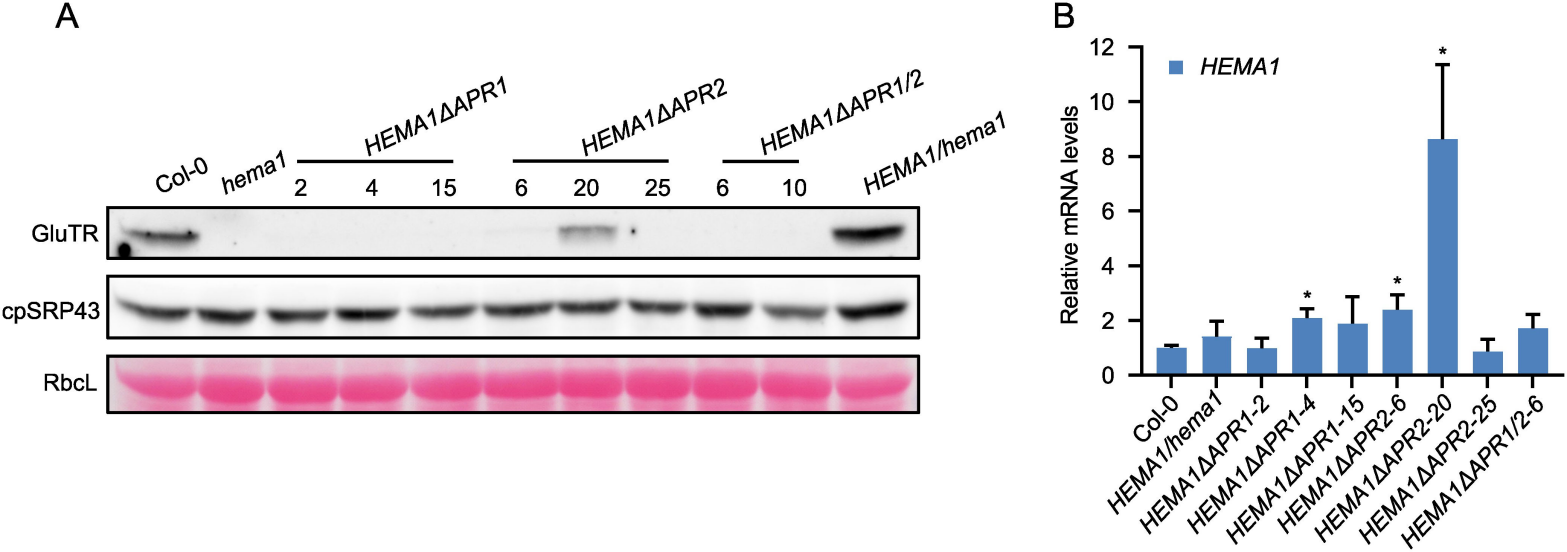
Deletion of APRs dramatically affects GluTR integrity. **(A)** Steady-state levels of GluTR and cpSRP43 in 16-day-old seedlings of Col-0, *hema1*, *HEMA1/hema1*, *HEMA1ΔAPR1*, *HEMA1ΔAPR2*, and *HEMA1ΔAPR1/2* grown under standard conditions were quantified by immunoblotting using the indicated antibodies. Ponceau S-stained RbcL is shown as a loading control. **(B)** Relative mRNA quantities of *HEMA1* in the indicated genotypes. *HEMA1* expression levels in all transgenic lines were normalized to those in Col-0 using *SAND* as the reference gene. All values are expressed as means ± s.d. (n = 3 independent biological repeats). Statistical analysis was performed using two-tailed Student’s *t*-tests. Asterisks indicate significant differences compared to *HEMA1* mRNA levels in Col-0: *P < 0.05.

Immunoblot analysis of GluTR in mutants expressing the APR-deficient GluTR revealed GluTR content below the detection level except in the *HEMA1ΔAPR2-20* mutant. In contrast, the levels of cpSRP43, which serves as the molecular chaperone for GluTR, remained comparable across all samples (**Figure 2A**). To exclude that low GluTR levels are due to transcriptional downregulation of the transgene, qRT-PCR analysis was conducted and revealed wild-type-like *HEMA1* levels in most transgenic lines, while *HEMA1ΔAPR2-20* exhibited elevated *HEMA1* transcript levels (**Figure 2B**). This suggests that GluTR is strongly destabilized with deleted APR motifs. In consistency with the non-detectable contents of GluTRΔAPR, the ALA synthesis rate was extremely low and hardly detectable (**Figure 1C**), although a weakly detectable mutant GluTR was observed in *HEMA1ΔAPR2-20* (**Figure 2A**). These findings indicate that APR deletion disrupts the integrity of GluTR, destabilizes the protein and, thus, impairs its in-planta activity.

It is important to note, however, that the two GluTR-APRs are situated within the N-terminal catalytic domain, a region crucial for the enzyme’s activity. Their deletion is likely to inevitably impact both the enzyme’s activity and the structural integrity of GluTR. The described approach of removing the APR fragments from the GluTR structure thus resulted in a very vulnerable and fragile protein. From these observations it can be deduced that the deletion of the small APR peptides disorganizes the structure of GluTR and the protein is immediately degraded. The deletion of the APRs therefore probably proved to be too disruptive to the structure of GluTR. These GluTR deletion mutants appear to be unsuitable for studying the functional role of APRs in GluTR.

### 3.3 Amino acid residues substitution in GluTR APRs

Based on the results of the first attempt to analyze the properties of APRs in GluTR by deleting them within the protein structure, we intend to modify insignificantly the APR sequence to a non-APR by only one conservative amino acid replacement in the motif. It was expected that the aggregation tendency of GluTR would be affected, but without the risk of significantly changing the three-dimensional conformation and thus the enzymatic activity. The TANGO algorithm (Fernandez-Escamilla et al., 2004) was applied to assess the aggregation tendency of the APRs after substitutions. As predicted, APRs are rich in aliphatic hydrophobic residues such as valine (Val), leucine (Leu), and isoleucine (Ile), as well as aromatic residues like phenylalanine (Phe), tyrosine (Tyr), and tryptophan (Trp) (Rousseau et al., 2006). Additionally, flanking charged residues and proline (Pro) residues act as “gatekeeper” residues that can alleviate aggregation (Reumers et al., 2009). In the case of GluTR, the two APRs were enriched with Val, Leu, and Ile.

**Figure 3:**
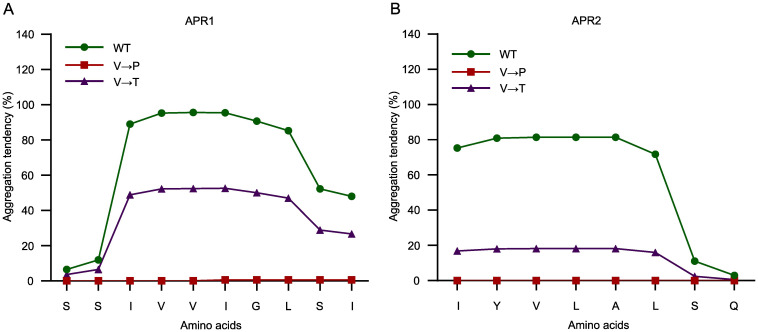
Amino acid residue substitutions in APR abolish aggregation tendency. The amino acid substitutions in APR1 **(A)** and APR2 **(B)** were analyzed using the TANGO algorithm. The original sequences (green), V→P substituted sequences (red), and V→T substituted sequences (purple) are plotted against their predicted aggregation tendencies.

With reference to previous APR studies (Richardson and Richardson, 2002; Betti et al., 2016), we hypothesized that substituting hydrophobic residues with Pro could reduce aggregation. Using the TANGO algorithm, the replacement of Val-99 or Val-151 with Pro in each of the two APRs was assessed to completely abolish the GluTR aggregation (**Figures 3A, B**). To further corroborate this concept, the aggregation tendency of GluTR by replacing Val with threonine (Thr) (V99T and V151T) in the two APRs was predicted. The side chains of Val and Thr are similar in size, but differ significantly in polarity. Val contains a hydrophobic methyl group (-CH3) and Thr contains a polar hydroxyl group (-OH) (**Supplementary Figure S2A**). Using the TANGO algorithm, it was predicted that the V→T substitutions could theoretically reduce the aggregation tendency of APR1 by 50% and that of APR2 by 60% (**Figures 3A, B**). These results suggest that amino acid substitutions can reduce the aggregation propensity of APRs, possibly without disrupting the overall structure of GluTR.

### 3.4 Generation and analysis of APRs point mutants

**Figure 4:**
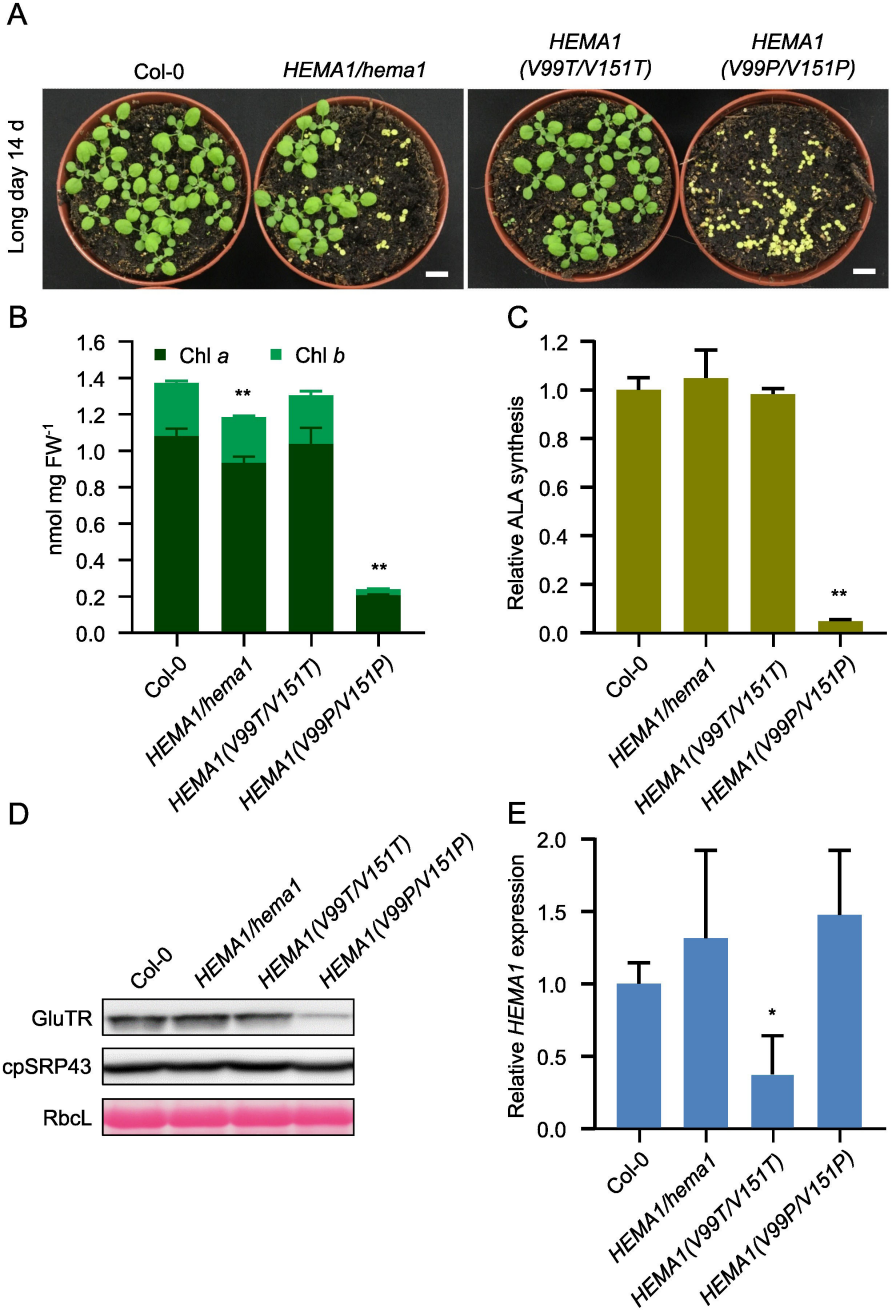
Characterization and analysis of the transgenic lines *HEMA1(V99T/V151T)* and *HEMA1(V99P/V151P)*. **(A)** Representative image of 14-day-old seedlings of Col-0, *HEMA1/hema1*, *HEMA1(V99T/V151T)*, and *HEMA1(V99P/V151P)* grown under standard conditions (16 h light/8 h dark, 100 μmol photons m^-2^ s^-1^). Scale bar: 1 cm. **(B, C)**, Chl content **(B)** and ALA synthesis rate **(C)** in 16-day-old seedlings of Col-0, *HEMA1/hema1, HEMA1(V99T/V151T)*, and *HEMA1(V99P/V151P)* grown under standard conditions. FW: fresh weight. **(D)** Steady-state levels of cpSRP43 and TBS proteins in 14-day-old Col-0 seedlings, *HEMA1/hema1*, *HEMA1(V99T/V151T)*, and *HEMA1(V99P/V151P)*, quantified by immunoblotting using the indicated antibodies. Ponceau S-stained RbcL is shown as a loading control. **(E)** Relative mRNA levels of *HEMA1* in the indicated seedlings, normalized to Col-0 levels using *SAND* as the reference gene. All values are expressed as means ± s.d. (n = 3 independent samples). Statistical analysis was performed using two-tailed Student’s *t*-tests. Asterisks indicate significant differences compared to Col-0: *P < 0.05, **P < 0.01.

We designed the APRs point mutants of the *HEMA1* sequence, cloned the mutant *HEMA1* sequences behind the *HEMA1* promoter, which was already available in a binary vector pJA1 variant, and transformed via *Agrobacterium tumefaciens* the Arabidopsis *hema1* mutant. The transgenic lines were designated as *HEMA1(V99P/V151P)* and *HEMA1(V99T/V151T)*. Sanger sequencing analyses confirmed the successful generation of these lines (**Supplementary Figure 2B**). The lines expressing *HEMA1(V99T/V151T)* displayed a growth phenotype similar to wild type, whereas *HEMA1(V99P/V151P)* exhibited yellow seedlings and significant growth retardation, resembling the *hema1* phenotype (**Figure 4A**). Accordingly, the Chl content in *HEMA1(V99P/V151P)* was dramatically decreased, while it remained unchanged in *HEMA1(V99T/V151T)* (**Figure 4B**). The leaf phenotype of these lines expressing either *HEMA1(V99P/V151P)* or *HEMA1(V99T/V151T)* corresponds to modified Chl accumulation and biosynthesis. It is hypothesized that expression of GluTR(V99P/V151P) most likely compromises the ALA synthesis rate, as the *HEMA1(V99T/V151T)* line showed an ALA synthesis rate comparable to that of the wild type, whereas the rate was drastically reduced in the *HEMA1(V99P/V151P)* line (**Figure 4C**).

qRT-PCR and immunoblotting were employed to examine the transcript and protein levels of GluTR variants in the *HEMA1(V99P/V151P)* and *HEMA1(V99T/V151T)* lines. The GluTR level was significantly reduced in *HEMA1(V99P/V151P*), while the cpSRP43 level remained unchanged, similar to the control (**Figure 4D**). Notably, the *HEMA1* transcript level was similar to that of wild type (**Figure 4E**), indicating that the V→P mutation diminished GluTR stability. The impaired steady-state level of GluTR(V99P/V151P) in *HEMA1(V99P/V151P)* correlated with the vanishingly low rate of ALA synthesis (**Figure 4C**), suggesting that the V→P mutations drastically impair the stability of the mutant GluTR and likely do not directly affect the enzymatic activity. Collectively, these findings suggest that the V→P mutation negatively impacts the conformational integrity of GluTR.

### 3.5 GluTR(V99T/V151T) enhances the stability of GluTR

**Figure 5:**
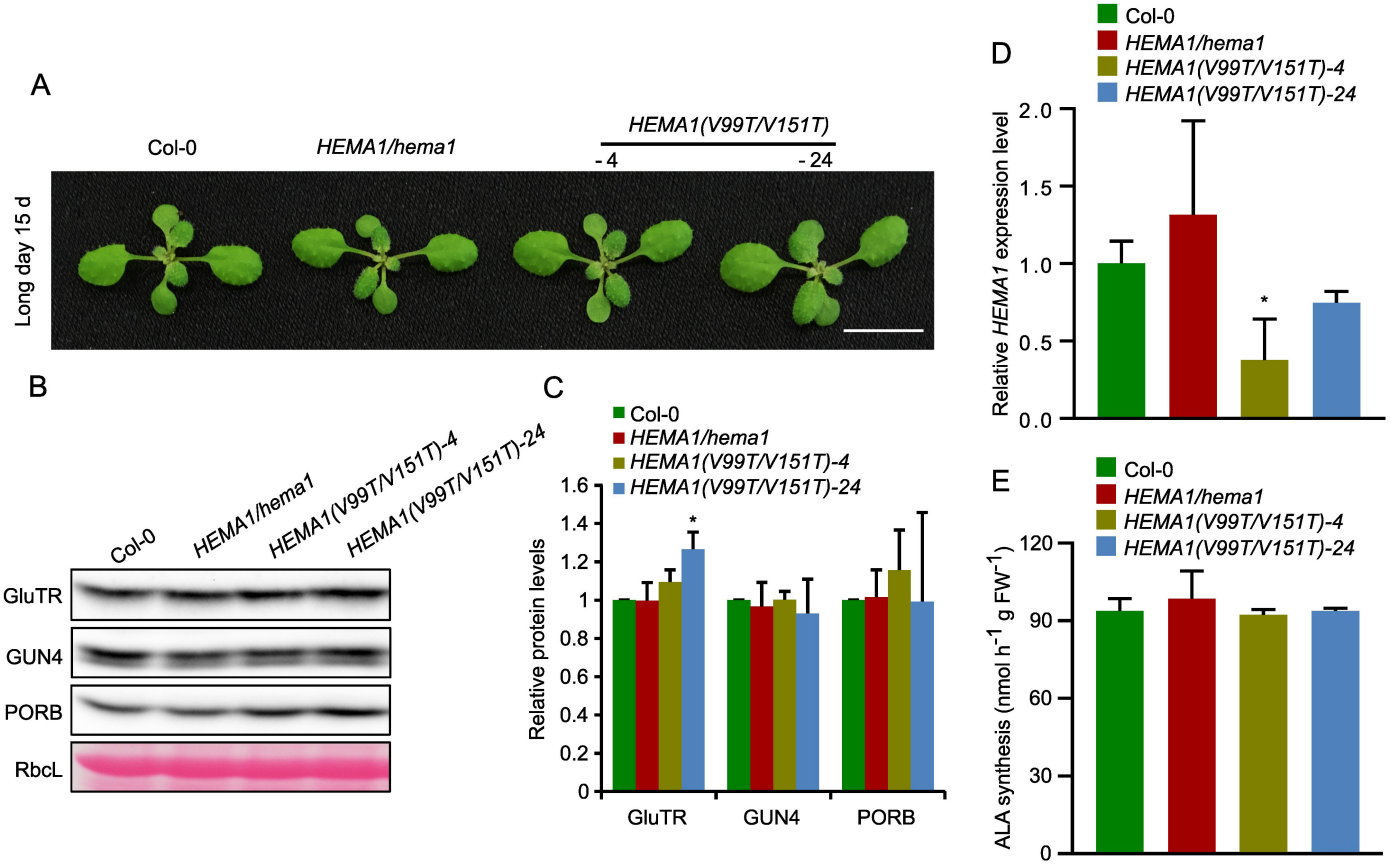
Analysis of transcript and protein levels of GluTR in *HEMA1(V99T/V151T)*. **(A)** Representative image of 15-day-old plants of Col-0, *HEMA1/hema1*, *HEMA1(V99T/V151T)-4*, and *HEMA1(V99T/V151T)-24* grown under standard conditions (16 h light/8 h dark, 100 μmol photons m^-2^ s^-1^). Scale bar: 1 cm. **(B)** Steady-state levels of GluTR, GUN4, and PORB in the indicated seedlings, quantified by immunoblotting using the indicated antibodies. Ponceau S-stained RbcL is shown as a loading control. **(C)** Semi-quantitative analysis of the immunoblots in panel **(B)** using ImageJ software (NIH). The relative amounts of GluTR, GUN4, and PORB in *HEMA1(V99T/V151T)-4* and *HEMA1(V99T/V151T)-24* were normalized to levels in Col-0. **(D)** Relative mRNA levels of *HEMA1* in Col-0, *HEMA1/hema1*, *HEMA1(V99T/V151T)-4 and HEMA1(V99T/V151T)-24* seedlings. Gene expression levels were calculated relative to Col-0 using *SAND* as the reference gene. **(E)** ALA synthesis rates in *HEMA1(V99T/V151T)-4 and HEMA1(V99T/V151T)-24* seedlings grown under standard growth conditions. The data are plotted as means ± s.d. (*n* = 3 independent biological repeats). Statistical analysis was performed using two-tailed Student’s *t*-tests. Asterisks indicate significant differences compared to Col-0: *P < 0.05.

We selected two lines of *HEMA1(V99T/V151T)*, which show a wild-type-like phenotype (**Figure 5A**). Although the transcript level of *HEMA1* in *HEMA1(V99T/V151T)-24* was comparable to that of wild type (**Figure 5D**), GluTR(V99T/V151T) accumulated more than in wild type (27%). In contrast, as control, the levels of the other TBS proteins, GUN4 and PORB, remained unchanged (**Figures 5B, C**). An almost 50% reduced transcript level of *HEMA1(V99T/V151T)* in line #4 correlates with a GluTR(V99T/V151T) content, which is similar to wild type. These observations would be consistent with a proposed improved stability of the V→T mutant of GluTR compared to wild type (**Figures 5B, C**). However, the ALA synthesis rates in both lines *HEMA1(V99T/V151T)-4* and *HEMA1(V99T/V151T)-24* were similar to those of wild type. (**Figure 5E**), suggesting that V→T substitutions do not affect ALA synthesis rates *in vivo*. Since potentially elevated levels of GluTR are detectable at least in the *HEMA1(V99T/V151T)-24* line, it is conceivable that due to the complex post-translational control of ALA synthesis, other factors may also limit the overall rate of ALA synthesis, which consists of the reduction step of activated glutamate by GluTR and the transamination reaction by GSAT (Sinha et al., 2022). From the steady-state level of the GluTR(V99T/V151T) variants it can be deduced that a slightly increased stability of GluTR was achieved by the amino acid substitutions in the APRs.

### 3.6 Reduced *in vitro* aggregation of GluTR(V99T/V151T), but its unchanged assembly in protein complexes

**Figure 6:**
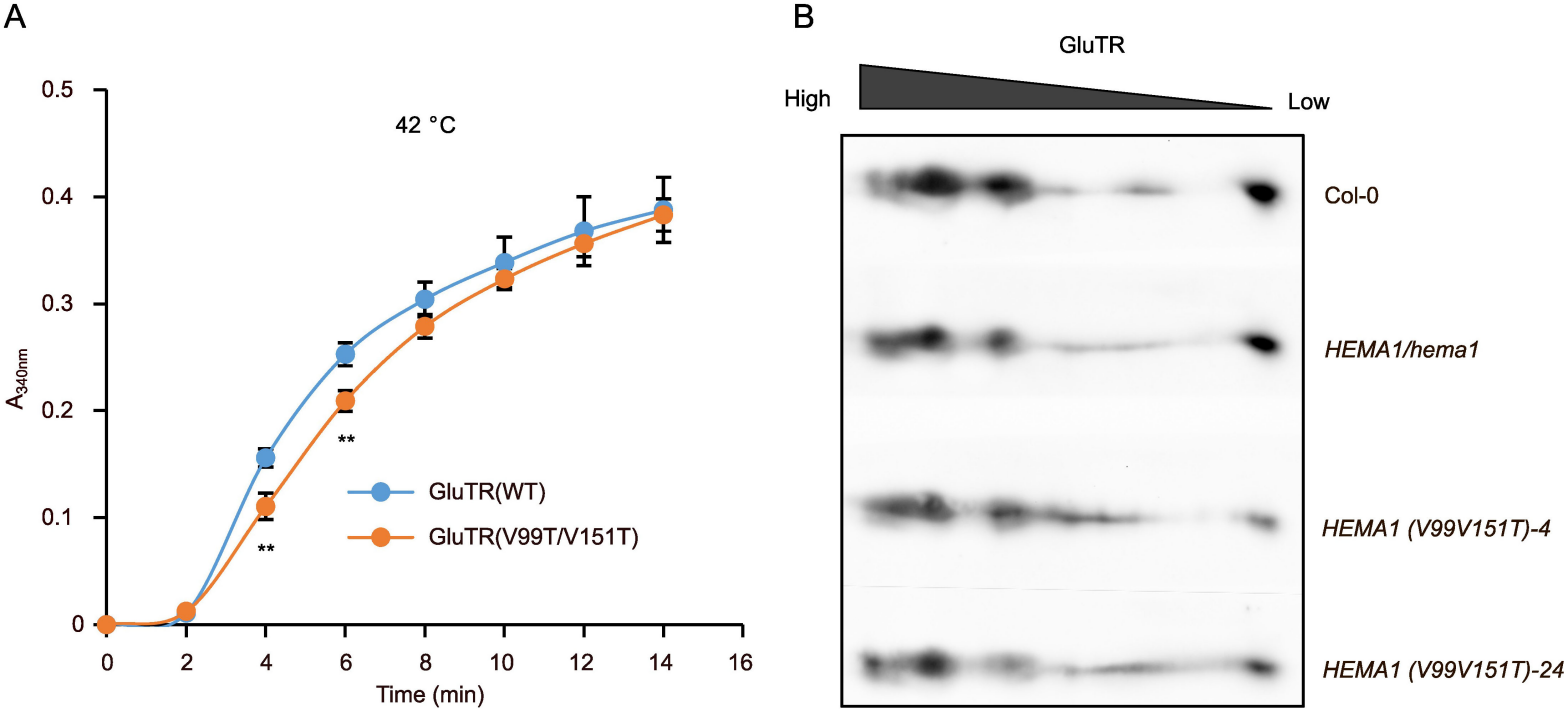
Aggregation and oligomerization analyses of GluTR in *HEMA1(V99T/V151T)*. **(A)** Scattering assays to evaluate the heat-induced aggregation of GluTR(WT) and GluTR(V99T/V151T). A_340 nm_ was measured at 2-min intervals to quantify turbidity. Thermal aggregation of His-GluTR(WT) and His-GluTR(V99T/V151T) (2 µM) were examined for 14 min at 42°C. Data are plotted as means ± s.d. (*n* = 3 independent biological repeats). Statistical analysis was performed using two-tailed Student’s *t*-tests. Asterisks indicate significant differences compared to GluTR(WT) aggregates: **P < 0.01. **(B)** Two-dimensional BN-SDS-PAGE analysis of GluTR allocation in chloroplasts isolated from Col-0, *HEMA1/hema1*, *HEMA1(V99T/V151T)-4* and *HEMA1(V99T/V151T)-24*. GluTR monomers and oligomers were detected by immunoblotting.

Since the TANGO algorithm predicts a tendency of decreased aggregation of GluTR(V99T/V151T), the protein was examined *in vitro* and *in vivo* to determine whether the V→T substitutions affect GluTR aggregation and/or oligomerization. We expressed and purified the recombinant GluTR(V99T/V151T) and wild-type GluTR (**Supplementary Figure S3**) and performed the light-scattering assays to examine the formation of GluTR aggregates *in vitro* at 42°C. The GluTR(V99T/V151T) showed a slight decrease in heat-induced aggregation by 18–30%, particularly at the 4–6 min time point compared to the wild-type GluTR (**Figure 6A**). However, it cannot be completely ruled out that these minor differences may also be due to a higher proportion of protein impurities in the purified GluTR(V99T/V151T) sample compared to the wild-type preparation (**Supplementary Figure S3**). Nonetheless, these findings also imply that the V→T substitutions enhance the stability of GluTR by inhibiting its tendency to aggregate.

Additionally, we conducted two-dimensional BN-PAGE to examine the oligomerization of GluTR in *HEMA1(V99T/V151T)*, *HEMA1/hema1*, and wild-type samples. As illustrated in **Figure 6B**, the immunoreactive GluTR was present in similar amounts in the different protein complexes of the analyzed protein extracts of the transgenic line *HEMA1(V99T/V151T), HEMA1/hema1* and wild type, indicating that the accumulation of GluTR(V99T/V151T) in the protein complexes of different sizes was not significantly altered. Based on these *in vivo* and *in vitro* assays, GluTR(V99T/V151T) is assumed to contain slightly modified APRs that do not affect the assembly of GluTR in other protein complexes. These complexes may also contain other proteins or consist solely of GluTR oligomers.

### 3.7 The GluTR(V99T/V151T) variant does not increase thermostability of GluTR

**Figure 7:**
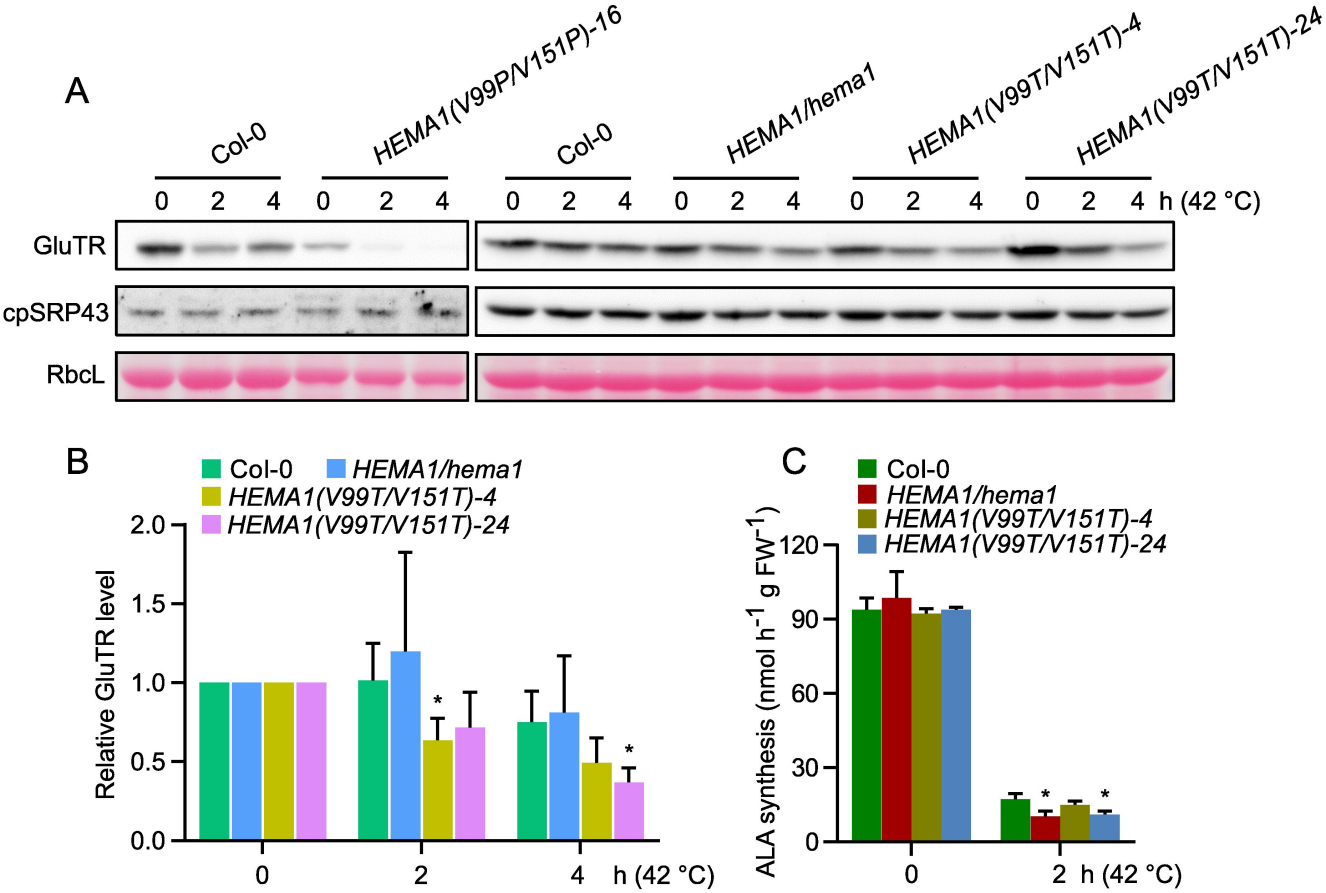
Amino acid substitutions in GluTR do not improve the thermostability. **(A)** Steady-state levels of GluTR and cpSRP43 in 16-day-old seedlings of Col-0, *HEMA1/hema1*, *HEMA1(V99P/V151P)-16*, *HEMA1(V99T/V151T)-4*, and *HEMA1(V99T/V151T)-24*, before (0 h at 42°C) or after 2–4 hours of heat treatment (2–4 h at 42°C), detected by immunoblotting using the indicated antibodies. Ponceau S-stained RbcL is shown as a loading control. **(B)** Semi-quantitative analysis of the immunoblots in panel **(A)** using ImageJ software (NIH). The relative amounts of GluTR in *Col-0*, *HEMA1/hema*, *HEMA1(V99T/V151T)-4*, *and HEMA1(V99T/V151T)-24* were normalized to their respective levels at the 0 h. **(C)** ALA synthesis rates in Col-0, *HEMA1/hema1*, *HEMA1(V99T/V151T)-4*, and *HEMA1(V99T/V151T)-24* under standard conditions (42°C, 0 h) and heat shock conditions (42°C, 2 h). Data are plotted as means ± s.d. [n = 4 independent biological repeats for panel **(B)**, and n = 3 independent biological repeats for panel **(C)**]. Statistical analysis was performed using two-tailed Student’s *t*-tests. Asterisks indicate significant differences compared to ALA synthesis rates in Col-0: *P < 0.05.

It is currently hypothesized that APRs potentially enhance the aggregation propensity of GluTR and heat shock treatment exacerbates its aggregation and compromise stability. Therefore, we asked whether the amino acid substitutions in the APRs could enhance the thermostability of GluTR. We subjected seedlings from Col-0 and the *HEMA1/hema1*, *HEMA1(V99P/V151P)*, and *HEMA1(V99T/V151T)* lines to a heat shock treatment and determined the GluTR levels after a 2- and 4-hour-treatment at 42°C. We found that the GluTR variant in *HEMA1(V99P/V151P)* lines degraded more drastically than the wild type, while GluTR(V99T/V151T) in *HEMA1(V99T/V151T)* also showed a faster degradation compared to the wild type. In contrast, the level of the chaperone protein cpSRP43 remained unchanged (**Figures 7A, B**). Further analysis of ALA synthesis rates under heat shock confirmed the instability of the mutant GluTR in *HEMA1(V99T/V151T)* (**Figure 7C**), suggesting that neither the V→P nor V→T mutations improve GluTR thermostability under heat shock.

## 4 Discussion

### 4.1 The V→T mutations slightly improve the stability of GluTR

Our studies showed for the first time for a plant-specific enzyme that the APRs, which are situated in the catalytic domain at the N-terminus of GluTR, contribute substantially to the functional 3D structure and conformation of GluTR, which are important for the integrity and enzymatic activity.

It was assessed that the substitution of Val99 and Val151 with Pro completely abolished the aggregation propensity of GluTR, whereas the substitutions with threonine reduced it by 50% in APR1 and 60% in APR2, respectively (**Figure 3**). These theoretical assessments led to experimental *in vivo* and *in vitro* studies with both purified recombinant substitution mutant GluTR(V99T/V151T) or transgenic lines in the *hema1* background that express *HEMA1(V99P/V151P)* or *HEMA1(V99T/V151T).*

Although the transcription level of *HEMA1* in *HEMA1(V99P/V151P)* was similar to wild type (**Figure 4E**), the GluTR level was significantly decreased (**Figure 4D**), suggesting that the V→P substitutions severely compromised GluTR stability. In addition, phenotypic analysis revealed that the transgenic Arabidopsis line *HEMA1(V99P/V151P)* resembled the *hema1* knockout mutant (**Figure 4A**). As the APRs are placed at the N-terminus of GluTR (aa95–aa156), it is hypothesized that these two APRs play a critical role in maintaining the structural integrity of GluTR.

In continuation, *in vitro* scattering assays revealed that the aggregation of GluTR(V99T/V151T) was only marginally reduced compared to wild-type GluTR (**Figure 6A**). As a result, the GluTR(V99T/V151T) level was shown to be slightly increased by 27% in one representative *HEMA1(V99T/V151T)* line (**Figures 5B, C**), suggesting that the mutation V→T likely leads to increased stability of GluTR and reduced aggregation tendency. However, despite the increased accumulation of GluTR, no significant increase in the ALA synthesis rate was observed (**Figure 5E**), indicating a lower GluTR activity caused by the V→T mutations. A possible explanation for this observation lies in the location of the APRs in the catalytic domain of GluTR. However, it is also not excluded that the elevated total GluTR content did not necessarily increase the soluble amount of GluTR (e.g. the portion of GluTR, which is not associated with the thylakoid membrane), as soluble GluTR content is considered to correlate with the ALA synthesis rates (Schmied et al., 2018; Hou et al., 2019).

Although the V→T mutation improves the stability of GluTR, it does not enhance its thermostability (**Figures 7A, B**). One possible explanation is that the V→T mutation may impair the interaction between cpSRP43 and GluTR. While a lower cpSRP43-GluTR affinity might be sufficient to protect GluTR under normal conditions, it may not provide enough protection under heat shock conditions. Then, the GluTR variant could become more susceptible to degradation by Clp protease under heat stress. Furthermore, it is not excluded that other unknown chaperones, which may collaborate with cpSRP43 for the stability of GluTR under stress conditions, are unable to fully compensate for this impaired interaction.

In conclusion, the V→T mutation apparently tends to reduce the aggregation of GluTR and improve the stability, but not activity of GluTR in planta. Since the aggregation tendency and protein stability of the (V→T)GluTR variant were only slightly improved, it is not excluded that further strategic optimization of amino acid substitutions could more effectively reduce aggregation and improve the stability of GluTR.

### 4.2 Structural alignments indicated that the APR structures are crucial for maintaining the structural integrity of GluTR

Using AlphaFold, we predicted the structures of the APR-deleted GluTR and compared them with the wild-type GluTR (**Supplementary Figure S4**). The deletion of APRs resulted in significant structural changes in the entire GluTR protein, particularly in the catalytic domain (black arrows), the RED domain (black solid triangles), and the dimerization domain (black solid circles). Focusing on the catalytic domain, which is crucial for enzymatic activity of GluTR, we observed structural changes at two and four positions following the deletion of APR1 (**Supplementary Figure S4B**) and APR2 (**Supplementary Figure S4C**), respectively, and at five positions when both APR1 and APR2 were simultaneously deleted (**Supplementary Figure S4D**). This suggests that APR2 may play a more critical role than APR1 in maintaining the structural integrity of GluTR. Notably, while previous *in vitro* studies (Wang et al., 2018) demonstrated that APR deletions enhance solubility of N-terminal GluTR fragment (GluTR-N) — evidenced by significantly reduced aggregation of GluTR-NΔAPR2 compared to GluTR-NΔAPR1 — our *in vivo* data tell a different story. In the cellular environment, the deletion of APRs apparently leads to severe disruption of the overall structure of GluTR resulting in an unstable protein that is rapidly degraded by unknown protein quality control mechanisms.

Structural alterations were observed not only in the APR region but also in the RED and dimerization domains. However, these changes in RED and dimerization domains may not explain the destabilization and loss of GluTR activity following APR deletion, as similar structural alterations were also observed in the GluTR(V99T/V151T) structure (**Supplementary Figure S4F**). In GluTR(V99P/V151P), the catalytic domain exhibited notable structural changes in the APR1 region and its adjacent β-sheet (black arrow) (**Supplementary Figure S4E**), which could account for the compromised stability and activity of GluTR. In contrast, the APRs structure in GluTR(V99T/V151T) remained identical to wild type, suggesting that the V→T mutation does not impact the structure of the catalytic domain. This observation could explain why the integrity of GluTR remains unaffected in the *HEMA1(V99T/V151T)* line.

Moreover, these results support the idea that the structural integrity of GluTR critically depends on the presence of the APR motifs, and that their deletion leads to severe disruption of the overall structure. As a result, GluTR becomes extremely unstable, is quickly degraded, and loses its enzymatic activity. Overall, this would highlight the crucial role of APRs in maintaining GluTR stability and function.

### 4.3 APRs in other TBS proteins

GluTR, CHLH and GUN4, the substrate-binding protein and the positive regulator of MgCh, as well as the B-isoform of POR (PORB) interact with the chaperone cpSRP43 (Wang et al., 2018; Ji et al., 2021, 2023). It has been suggested that these interactions prevent the aggregation and degradation of these strongly regulated enzymes. As depicted in the **Supplementary Figures S5A–C** [GluTR, see Figure 7A in (Wang et al., 2018)], CHLH, GUN4, and PORB each have 21, 2 and 5 predicted APRs, respectively. Notably, CHLH harbors 9 APRs with aggregation tendencies exceeding 80%, GUN4 has a single APR with an aggregation tendency of 72%, while the highest aggregation tendency in PORB is only 22%. Thus, it is suggested that CHLH and GUN4 can form aggregates more easily upon unfolding and misfolding than PORB.

Sequence analyses of these APRs from all different TBS enzymes indicate no obvious similarities in their amino acid sequences, and their lengths differ between long and short peptide motifs (**Supplementary Table S3**). However, previous studies have indicated that APRs are significantly enriched in aliphatic hydrophobic residues, such as Val, Ile and Leu (Rousseau et al., 2006). We also found that these three amino acid residues are enriched in the APRs of GluTR, CHLH, GUN4 and PORB (**Supplementary Table S3**). Furthermore, we attempted to reveal the similarities of APR positions and functions in the three-dimensional structures of these TBS proteins by using the crystal structures of Arabidopsis GluTR-GBP complex, GUN4 and NADPH-Pchlide membrane-bound PORB (Zhao et al., 2014; Hu et al., 2021; Nguyen et al., 2021), while for CHLH the structure of the cyanobacterial protein was available to us (Zhang et al., 2019). We used AlphaFold 3.0 to predict the three-dimensional structure of CHLH. The protein structures are displayed for GluTR (**Supplementary Figure S5D**), CHLH (**Supplementary Figure S5E**), GUN4 (**Supplementary Figure S5F**) and PORB (**Supplementary Figure S5G**), all the APRs were highlighted in red. We found that APRs in GluTR and PORB form β-sheets and are buried in the proteins. In contrast, GUN4 lacks any β-sheet in its crystal structure, and the APRs of GUN4 are found in two different α-helices and exposed on the surface. For CHLH, the APRs are distributed between β-sheets and α-helices in roughly equal proportions, with the majority of these regions being buried inside the protein.

The different localizations of the APRs have potential functional implications. For proteins such as GluTR and PORB, the APRs buried within the protein may minimize aggregation less under standard/normal conditions, but perhaps during translation into nascent proteins or prior to refolding of the mature protein after import into the plastid. In addition, these APRs may be exposed under stress, requiring chaperone intervention. The surface-exposed APRs in GUN4 may contribute to protein aggregation upon misfolding, requiring more robust or targeted chaperone binding. These observations suggest that the interaction with cpSRP43 — and possibly other chaperones — must be finely tuned depending on the structural context of the APRs. In other words, the nature and location of APRs may determine not only the intrinsic aggregation propensity of each TBS protein, but also the extent and type of chaperone support required to maintain their functional conformations, especially under stress conditions. Therefore, understanding the interplay between APR properties and chaperone interactions is crucial for elucidating the mechanisms by which TBS proteins in plants achieve and maintain their proper folding, stability and activity.

### 4.4 cpSRP43 may protect the unfolded enzymes of tetrapyrrole biosynthesis by masking their APRs

The nuclear-encoded TBS proteins are synthesized as precursor proteins in the cytosol before they are translocated through the translocon complexes in the outer and inner envelope membranes (TOC-TIC complexes) into the stroma and to their final destinations. During this import through the outer and inner envelope membrane, these proteins are subjected to unfolding before they are refolded inside the plastids (Rochaix, 2022; Sun and Jarvis, 2023). In the unfolded state their surface-exposed APRs can promote protein aggregation, which is most likely even more enhanced during elevated temperature. It is therefore proposed that the highly controlled enzymes of TBS require special chaperones that protect these enzymes during their unfolded state, i.e., before refolding.

Previous *in vitro* studies have revealed that cpSRP43 binds to the APR-containing N-terminus of GluTR and prevents GluTR aggregation (Wang et al., 2018). Since cpSRP43 also protects the APR-harboring TBS proteins CHLH, GUN4, and PORB (Ji et al., 2021, 2023), we assume that cpSRP43 also protect these enzymes by covering their APRs. Since most of the APRs of GluTR, CHLH, and PORB are buried within the proteins (**Supplementary Figure S5**), cpSRP43 likely acts on these proteins, when they are unfolded. We speculate that assembly in aggregates and the prevention of aggregates belong to mechanisms of posttranslational modification of TBS proteins, to maintain protein homeostasis for always the precise amounts of these proteins and their adequate activity. Both features are strongly affected through adverse environmental conditions, such as elevated temperature. Therefore, functional depletion of these proteins could be a suitable mechanism under heat stress conditions (Wang et al., 2018; Ji et al., 2021). At present, it would be too speculative to evaluate the role of APRs of GUN4 located at the protein surface. As a positive regulator, GUN4 might have specific needs for multi-step post-translational control, including aggregation, stability or protein degradation. It remains of great interest to decipher the activity and steady state of GUN4. Future studies investigating the functional role of APRs in TBS proteins and their interaction with chaperones such as cpSRP43 may deepen our understanding of the biochemical mechanisms that control protein aggregation and disaggregation in the TBS pathway.

### 4.5 A final perspective: can point mutations of APR be a potential strategy to improve the protein stability of GluTR and other TBS enzymes?

In this study, the V→T mutation resulted in only 18–30% reduced GluTR aggregation in the *in-vitro* scattering assays (**Figure 6A**). This modest decrease in aggregation is associated with a 27% increase in the stability of GluTR(V99T/V151T) *in vivo* (**Figures 5B, C**), suggesting a positive correlation between the *in vivo* enhanced stability of GluTR and the reduced *in vitro* aggregation. These results support the potential of point mutations in APRs as a strategy to improve the functional integrity of GluTR. Given the central role of GluTR as the rate-limiting enzyme of TBS, which is crucial for chlorophyll production and photosynthetic efficiency, improving its stability could significantly increase TBS efficiency and ultimately plant productivity.

However, several limitations must be acknowledged. First, the observed slight decrease in aggregation suggests that point mutations alone may not fully resolve protein instability, especially for GluTR with its complex structure, unusual enzyme mechanism, intricately regulated dynamics of interaction with the neighboring enzymes and regulators, and also the existing APRs. Second, this study initially used only the Arabidopsis protein as a proof of concept and focused exclusively on this protein within other upregulated TBS enzymes, which may limit the transferability of the results to other TBS enzymes or crop species that may have other regulatory or structural features. Furthermore, the impact of these mutations on other functional properties of GluTR, such as its catalytic activity or the interactions with other enzymes (GSAT) and regulators (such as GBP, FLU, TTP1 etc.) need to be further investigated.

Future research should focus on optimizing amino acid substitutions by integrating bioinformatics and structural biology tools and methods to predict and validate the most effective mutations that reduce aggregation while maintaining or improving enzymatic function. Furthermore, combining point mutations with other stabilization strategies, such as incorporating molecular chaperones and stabilizing ligands, could provide a more robust approach to improve protein stability. Extending these investigations to other TBS enzymes and different plant species will be crucial to translate these findings into practical improvements in TBS efficiency and overall plant productivity.

## Data Availability

The original contributions presented in the study are included in the article/**Supplementary Material**. Further inquiries can be directed to the corresponding authors.
