# Supplementary material for: Modification of aggregation-prone regions of Arabidopsis glutamyl-tRNA reductase leads to increased stability while maintaining enzyme activity
Source: Front Plant Sci. 2025 Mar 13;16:1556843. doi: 10.3389/fpls.2025.1556843 (PMC11969407; doi:10.3389/fpls.2025.1556843)
Supplement: Supplementary file 1 [file DataSheet1.pdf]

Supplementary Material

Modification of aggregation-prone regions of Arabidopsis glutamyl-tRNA reductase provides increased stability while preserving enzyme activity

Shuiling Ji[1,2*], Peng Wang[2,3,4], Bernhard Grimm[2*]

*** Correspondence:**
Shuiling Ji
shuiling.ji@ccnu.edu.cn
Bernhard Grimm
bernhard.grimm@rz.hu-berlin.de

1 Supplementary Figures and Tables

1.1 Supplementary Figures

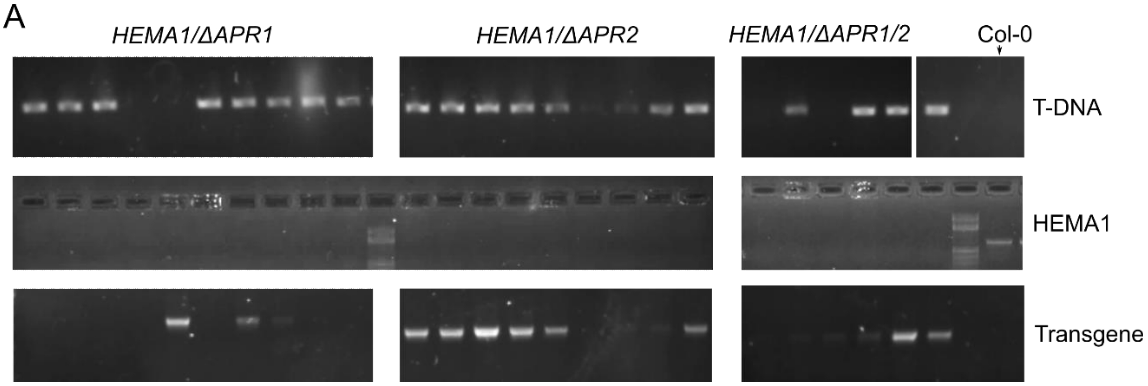

Figure S1. Genotypic analysis of APR deletion mutants. (A) PCR analysis of genomic DNA from Col-0, *HEMA1ΔAPR1*, *HEMA1ΔAPR2*, and *HEMA1ΔAPR1/2* confirmed the correct construct of the APR deletion mutants. T-DNA indicates amplification of the inserted T-DNA fragment. HEMA1 indicates amplification of the *HEMA*1 fragment, which is disrrupted by the T-DNA insertion in *hema1*. Transgene indicates amplification of the transformed APR deletion fragment. Primers used for genotypic analysis are listed in Table S2.

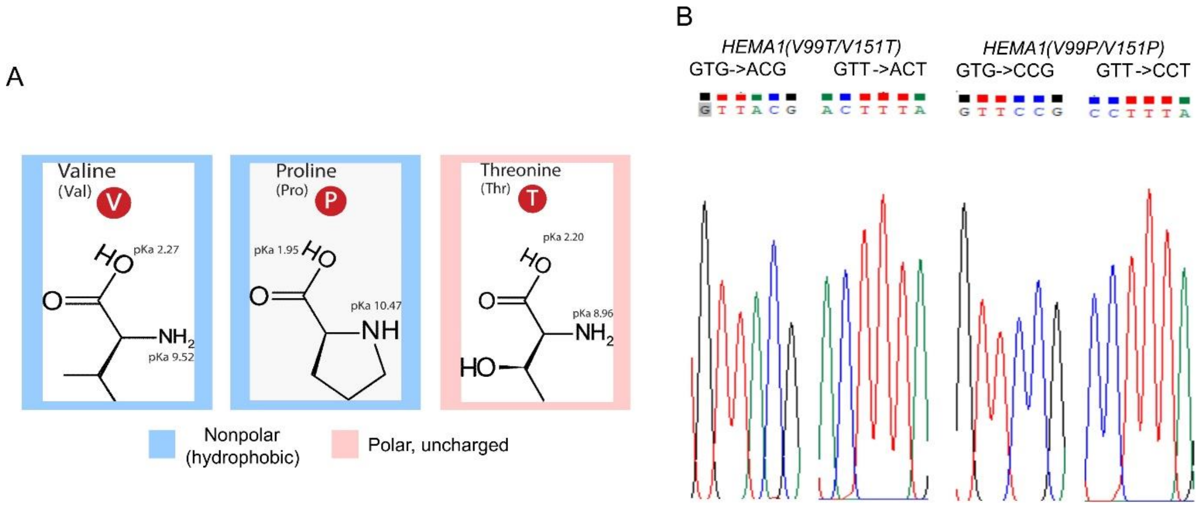

Figure S2. Characterization of the *HEMA1(V99T/V151T)* and *HEMA1(V99P/V151P)* transgenic lines. (A) Structural formula and hydrophobic properties of the amino acids Val (V), Pro (P), and Thr (T). **(B)** Sequencing confirmed successful transformation of the coding sequences for HEMA1(V99T/V151T) and HEMA1(V99P/V151P) in the homozygous transgenic lines *HEMA1(V99T/V151T)* and *HEMA1(V99P/V151P)*, respectively.

A

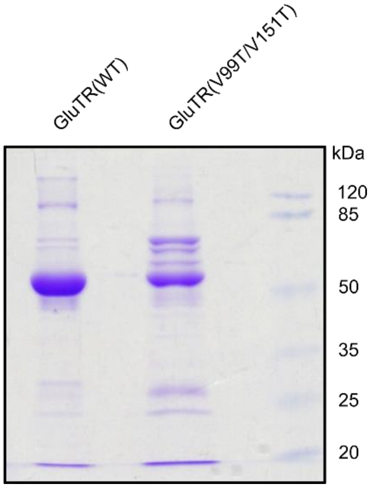

Figure S3. Recombinant proteins used in scattering assay. (A) Coomassie blue-stained purified recombinant GluTR and GluTR(V99T/V151T) proteins.

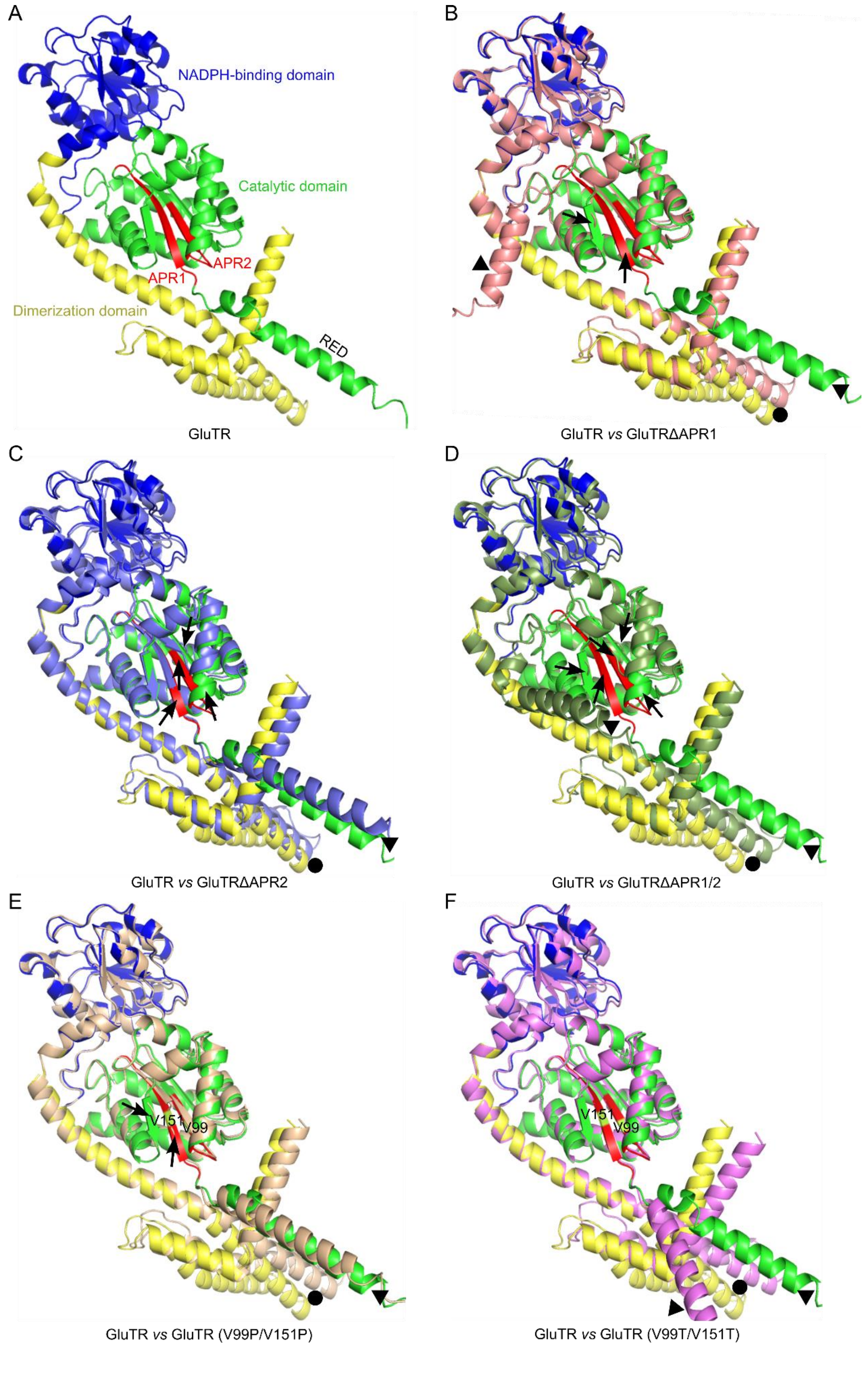
A
NADPH-binding domain
Catalytic domain
APR2
APR1
Dimerization domain
RED
GluTR
B
GluTR vs GluTRΔAPR1
C
GluTR vs GluTRΔAPR2
D
GluTR vs GluTRΔAPR1/2
E
V151
V99
GluTR vs GluTR (V99P/V151P)
F
V151
V99
GluTR vs GluTR (V99T/V151T)

Figure S4. Structural alignments of APR-mutated GluTRs with wild-type GluTR (WT). (A) Three-dimensional structure of wild-type GluTR(WT) predicted by AlphaFold 3, with functional domains labeled in different colors. The NADPH-binding domain is marked in blue, the catalytic domain including RED domain is marked in green, and the dimerization domain is marked in yellow. The two APRs are highlighted in red. **(B)**–**(F)**, Structural alignments of GluTR(WT) with the following variants: **(B)** GluTRΔAPR1 (light pink), **(C)** GluTRΔAPR2 (gray-blue), **(D)** GluTRΔAPR1/2 (olive green), **(E)** GluTR(V99P/V151P) (light orange), and **(F)** GluTR(V99T/V151T) (violet). Black solid triangles indicate structural variations in the N-terminal RED domain, black solid circles indicate variations in the dimerization domain, and black arrows indicate variations in the catalytic domain, including APRs. APRs are labeled in red, and V99 and V151 are marked in limon.

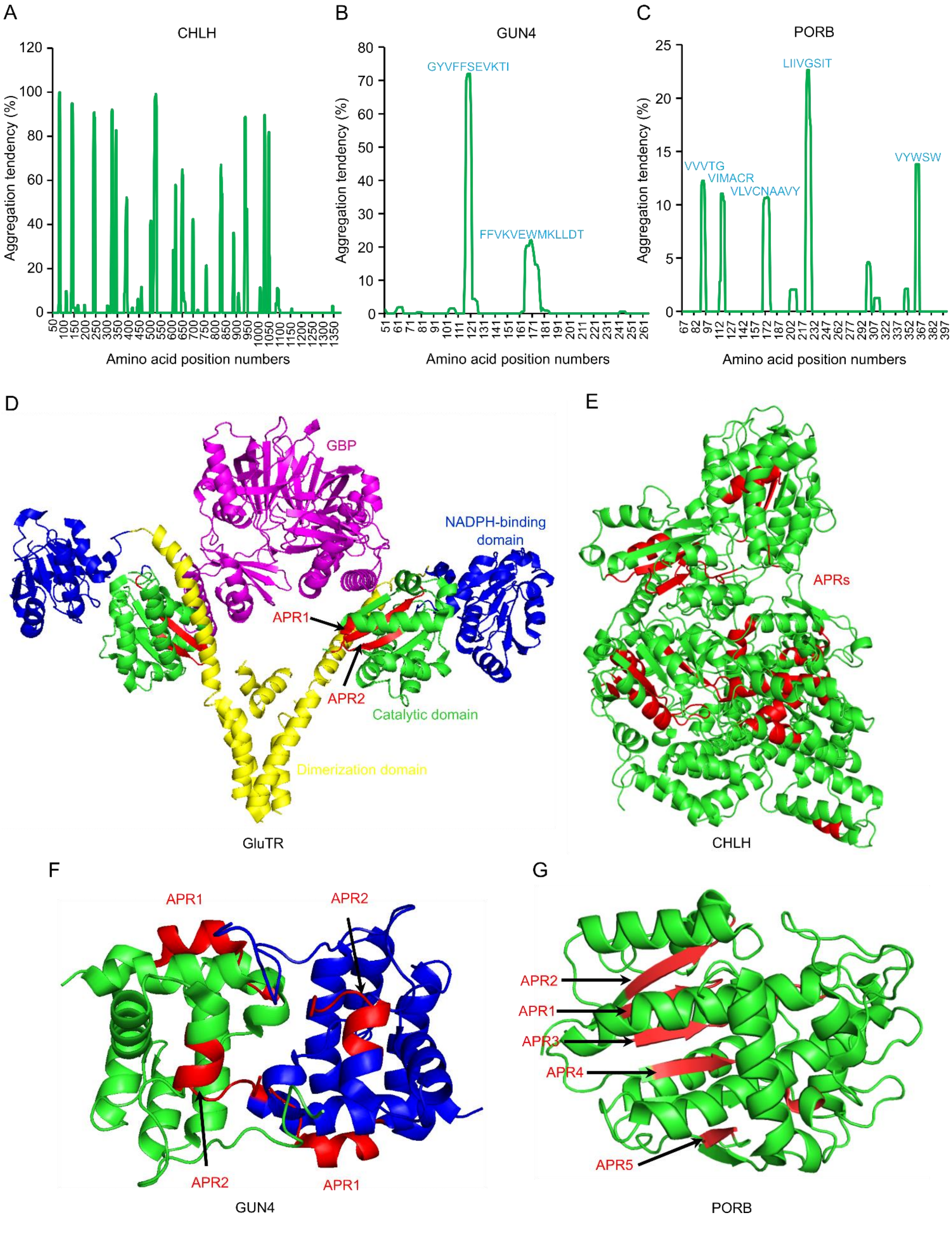

Figure S5. Aggregation-prone regions (APRs) predicted by TANGO in CHLH, GUN4, and PORB. (A)–(C), Aggregation tendencies of APRs in CHLH **(A)**, GUN4 **(B)**, and PORB **(C)**, the

sequences of APRs in GUN4 and PORB are highlighted in blue. **(D)–(G)**, Three-dimensional structures of GluTR-GBP dimer **(D)**, CHLH **(E)**, GUN4 dimer **(F)**, and PORB **(G)**, with APRs highlighted in red. The GBP dimer is labelled in purple, the NADPH-binding domain, catalytic domain, and dimerization domain are labeled in blue, red and yellow, respectively. CHLH and PORB monomers are marked in blue. In **(F)**, the red and blue colors represent the GUN4 monomers. The PDB IDs for GluTR-GBP heterodimer, GUN4 homodimer, and PORB are 4N7R, 7E2R, and 7JK9, respectively. The structure of CHLH was predicted using AlphaFold 3 (https://alphafold.ebi.ac.uk/). Protein structures were visualized with PyMOL software.

1.2 Supplementary Tables

Table S1. Genotypes used and analyzed in this study

**Line**	**Gene ID**	**Description**	**Source**
Col-0	-	Arabidopsis Columbia Wild type (WT)	-
HEMA1(V99P/V151P)	AT1G58290	*hema1* complementation line expressing GluTR(V99P/V151P)	This study
HEMA1(V99T/V151T)	AT1G58290	*hema1* complementation line expressing GluTR(V99T/V151T)	This study
HEMA1/hema1	AT1G58290	*hema1* heterozygous	(Apitz et al., 2014)
HEMA1ΔAPR1	AT1G58290	*hema1* complementation line expressing GluTR ΔAPR1	This study
HEMA1ΔAPR2	AT1G58290	*hema1* complementation line expressing GluTR ΔAPR2	This study
HEMA1ΔAPR1/2	AT1G58290	*hema1* complementation line expressing GluTR ΔAPR1/2	This study
hema1	AT1G58290	*HEMA1* knock-out mutant	(Apitz et al., 2014)

Table S2. List of primers used in this study

**Name**	**Sequences (5'->3')**	**Purpose**
SAND- Fw	AACTCTATGCAGCATTTGATCCACT	qRT-PCR
SAND- Rev	TGATTGCATATCTTTATCGCCATC	
HEMA1-Fw	TTGCTGCCAACAAAGAAGAC	
HEMA1-Rev	CCGTCTCCAATGAATCCCTC	
HEMA1_seq_Fw	TCAGATCCTTGCACAGGTGA	For analyses of transgenic lines *HEMA1ΔAPR1*, *HEMA1ΔAPR2*, *HEMA1ΔAPR1/2*
HEMA1_seq_Rev	TTGGAGACGTGACAGCAAAAT	
HEMA1_seq_Rev2	TCTCCTCACGGATAGCTGAAA	
TDNA_Fw	CTGGCGTAATAGCGAAGAGG	
HEMA1_Rev3	TAGGTTTACCCGCCAATATATCC	
pJAHemA1-Fw-LO	AAGGCGCGCCATGGCGGTTTCAAGTGCTTTCG	For generation of transgenic lines *HEMA1(V99P/V151P)*, *HEMA1(V99T/V151T)*
pJAHemA1-Rev-LO	CTCCTGCAGGTTACTTCTGTTGTTGTTCCGCC	
pJAHemA1V99P-Fw-LO	GAAGCAGTATTGTTCCGATTGGACTTAGTATTCACAC	
pJAHemA1V99P-Rev-LO	GTGTGAATACTAAGTCCAATCGGAACAATACTGCTTC	
pJAHemA1V151P-Fw-LO	CCGTATGGAGATTTATCCTTTAGCTTTATCTC	
pJAHemA1V151P-Rev	GAGATAAAGCTAAAGGATAAATCTCC	
pJAHemA1V99T-Fw-LO	GAAGCAGTATTGTTACGATTGGACTTAGTATTCACAC	
pJAHemA1V99T-Rev-LO	GTGTGAATACTAAGTCCAATCGTAACAATACTGCTTC	
pJAHemA1V151T-Rev	GAGATAAAGCTAAAGTATAAATCTCC	
pJAHemA1V151T-Fw-LO	CCGTATGGAGATTTATACTTTAGCTTTATCTC	
pJAHemA1V99T-Fw-LO	GAAGCAGTATTGTTACGATTGGACTTAGTATTCACAC	For expression of recombinant protein

pJAHemA1V99T-Rev-LO	GTGTGAATACTAAGTCCAATCGTAACAATACTGCTTC	GluTR(V99T/V151T)
pJAHemA1V151T-Rev	GAGATAAAGCTAAAGTATAAATCTCC	
pJAHemA1V151T-Fw-LO	CCGTATGGAGATTTATACTTTAGCTTTATCTC	

Table S3. Sequence analysis of the APRs in GluTR, CHLH, GUN4 and PORB

GluTR:

MAVSSAFVGCPKLETLLNHHNLSPSSSSSSSVSQTPLGLNGVRVLPKNNRTRRGLIQKARCELSASSDSASNAA
SISALEQLKNSAADRYTKERSSIVVIGLSIHTAPVEMREKLAIPEAEWPRAIAELCGLNHIEEAAVLSTCNRMEI
YVLALSQHRGVKEVTEWMSKTSGIPVSEICQHRFLLYNKDATQHIFEVSAGLDSLVLGEGQILAQVKQVVKV
GQGVNGFGRNISGLFKHAITVGKRVRTETNIASGAVSVSSAAVELALMKLPQSSNVSARMCVIGAGKMGKLVI
KHLMAKGCTKVVVVNRSEERVSAIREEMPGIEIIYRPLDEMLACASEADVVFTSTASETPLFLKEHVENLPQAS
PEVGGLRHFVDISVPRNVGSCVGEVETARVYNVDDLKEVVAANKEDRMRKAMEAQTIITEESTQFEAWRDSL
ETVPTIKKLRAYAERIRVAELEKCMSKMGDDINKKTTRAVDDLSRGIVNRFLHGPMQHLRCDGSDSRTLSETL
ENMHALNRMYGLEKDILEEKLKAMAEQQQK

CHLH:

MASLVYSPFTLSTSKAEHLSSLTNSTKHSFLRKKHRSTKPAKSFFKVKSAVSGNGLFTQTNPEVRRIVPIKRDNV
PTVKIVYVVLEAQYQSSLSEAVQSLNKTSRFASYEVVGYLVEELRDKNTYNNFCEDLKDANIFIGSLIFVEELAI
KVKDAVEKERDRMDAVLVFPSMPEVMRLNKLGSFSMSQLGQSKSPFFQLFKRKKQGSAGFADSMLKLVRTLP
KVLKYLPSDKAQDARLYILSLQFWLGGSPDNLQNFVKMISGSYVPALKGVKIEYSDPVLFLDTGIWHPLAPTM
YDDVKEYWNWYDTRRDTNDSLKRKDATVVGLVLQRSHIVTGDDSHYVAVIMELEARGAKVVPIFAGGLDFS
GPVEKYFVDPVSKQPIVNSAVSLTGFALVGGPARQDHPRAIEALKKLDVPYLVAVPLVFQTTEEWLNSTLGLHP
IQVALQVALPELDGAMEPIVFAGRDPRTGKSHALHKRVEQLCIRAIRWGELKRKTKAEKKLAITVFSFPPDKGN
VGTAAYLNVFASIFSVLRDLKRDGYNVEGLPENAETLIEEIIHDKEAQFSSPNLNVAYKMGVREYQDLTPYANA
LEENWGKPPGNLNSDGENLLVYGKAYGNVFIGVQPTFGYEGDPMRLLFSKSASPHHGFAAYYSYVEKIFKAD
AVLHFGTHGSLEFMPGKQVGMSDACFPDSLIGNIPNVYYYAANNPSEATIAKRRSYANTISYLTPPAENAGLYK
GLKQLSELISSYQSLKDTGRGPQIVSSIISTAKQCNLDKDVDLPDEGLELSPKDRDSVVGKVYSKIMEIESRLLP
CGLHVIGEPPSAMEAVATLVNIAALDRPEDEISALPSILAECVGREIEDVYRGSDKGILSDVELLKEITDASRGAV
SAFVEKTTNSKGQVVDVSDKLTSLLGFGINEPWVEYLSNTKFYRANRDKLRTVFGFLGECLKLVVMDNELGS
LMQALEGKYVEPGPGGDPIRNPKVLPTGKNIHALDPQAIPTTAAMASAKIVVERLVERQKLENEGKYPETIAL
VLWGTDNIKTYGESLGQVLWMIGVRPIADTFGRVNRVEPVSLEELGRPRIDVVVNCSGVFRDLFINQMNLLDR
AIKMVAELDEPVEQNFVRKHALEQAEALGIDIREAATRVFSNASGSYSANISLAVENSSWNDEKQLQDMYLSR
KSFAFDSDAPGAGMAEKKQVFEMALSTAEVTFQNLDSSEISLTDVSHYFDSDPTNLVQSLRKDKKKPSSYIAD
TTTANAQVRTLSETVRLDARTKLLNPKWYEGMMSSGYEGVREIEKRLSNTVGWSATSGQVDNWVYEEANST
FIQDEEMLNRLMNTNPNSFRKMLQTFLEANGRGYWDTSAENIEKLKELYSQVEDKIEGIDR

GUN4:

MATTNSLHHHHHSSPSYTHHRNNLHCQSHFGPTSLSLKQPTSAATFSLICSASSTSSSTTAVSAVSTTNASATTA
ETATIFDVLENHLVNQNFRQADEETRRLLIQISGEAAVKRGYVFFSEVKTISPEDLQAIDNLWIKHSDGRFGYSV
QRKIWLKVKKDFTRFFVKVEWMKLLDTEVVQYNYRAFPDEFKWELNDETPLGHLPLTNALRGTQLLKCVLS
HPAFATADDNSGETEDELNRGVAVAKEQAGVGADKRVFKTNYSF

PORB:

MALQAASLVSSAFSVRKDAKLNASSSSFKDSSLFGASITDQIKSEHGSSSLRFKREQSLRNLAIRAQTAATSSPT
VTKSVDGKKTLRKGNVVVTGASSGLGLATAKALAETGKWNVIMACRDFLKAERAAKSVGMPKDSYTVMHL
DLASLDSVRQFVDNFRRTETPLDVLVCNAAVYFPTAKEPTYSAEGFELSVATNHLGHFLLARLLLDDLKKSDY
PSKRLIIVGSITGNTNTLAGNVPPKANLGDLRGLAGGLNGLNSSAMIDGGDFDGAKAYKDSKVCNMLTMQEF
HRRFHEETGVTFASLYPGCIASTGLFREHIPLFRALFPPFQKYITKGYVSETESGKRLAQVVSDPSLTKSGVYWS
WNNASASFENQLSEEASDVEKARKVWEISEKLVGLA

The transit peptides are labeled in red, and the APRs are labeled in blue. The transit peptides were predicated using the TargetP-2.0 algorithm or downloaded from UniProt.

References

Apitz, J., Schmied, J., Lehmann, M.J., Hedtke, B., and Grimm, B. (2014). GluTR2 complements a hema1 mutant lacking glutamyl-tRNA reductase 1, but is differently regulated at the post-translational level. *Plant Cell Physiol* 55(3)**,** 645-657. doi: 10.1093/pcp/pcu016.

2 Original image files for the blots and gels

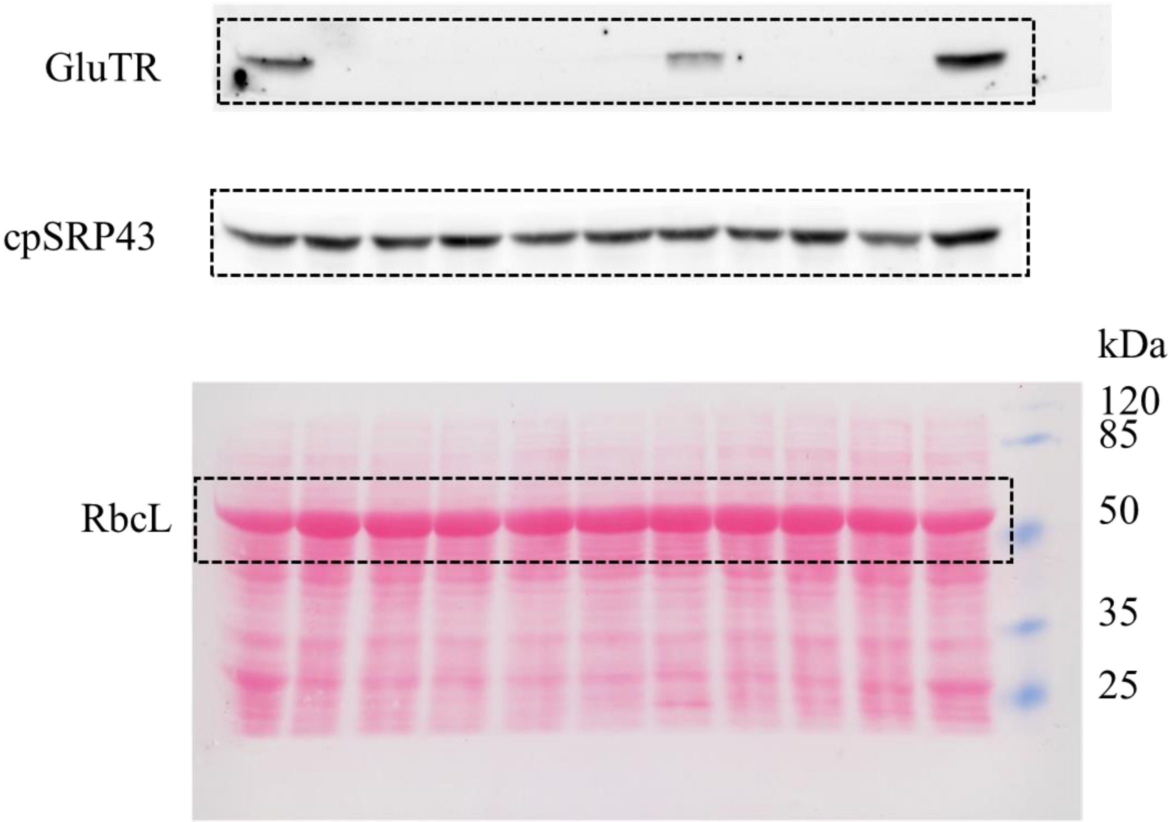

Original blots for the Figure 2A

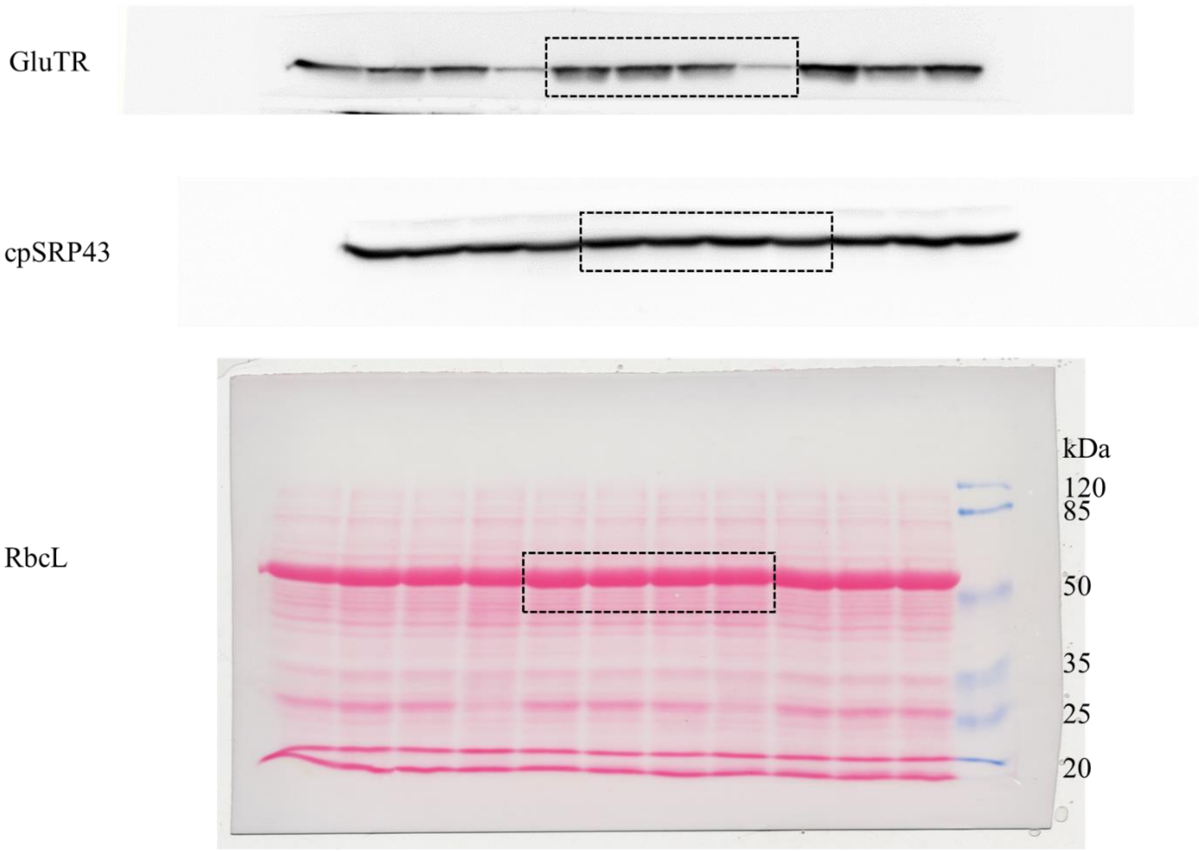

Original blots for the Figure 4D

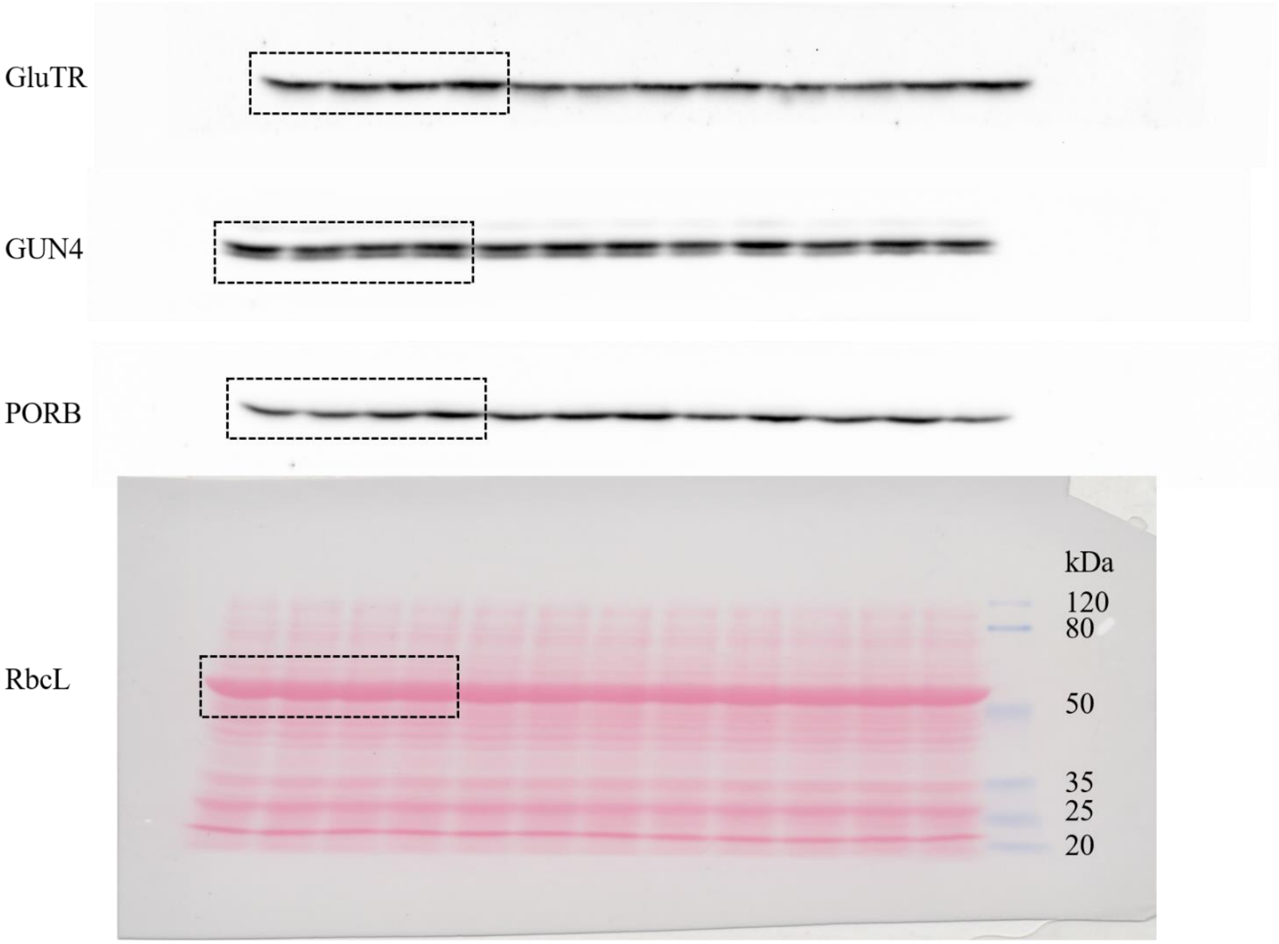

Original blots for the Figure 5B

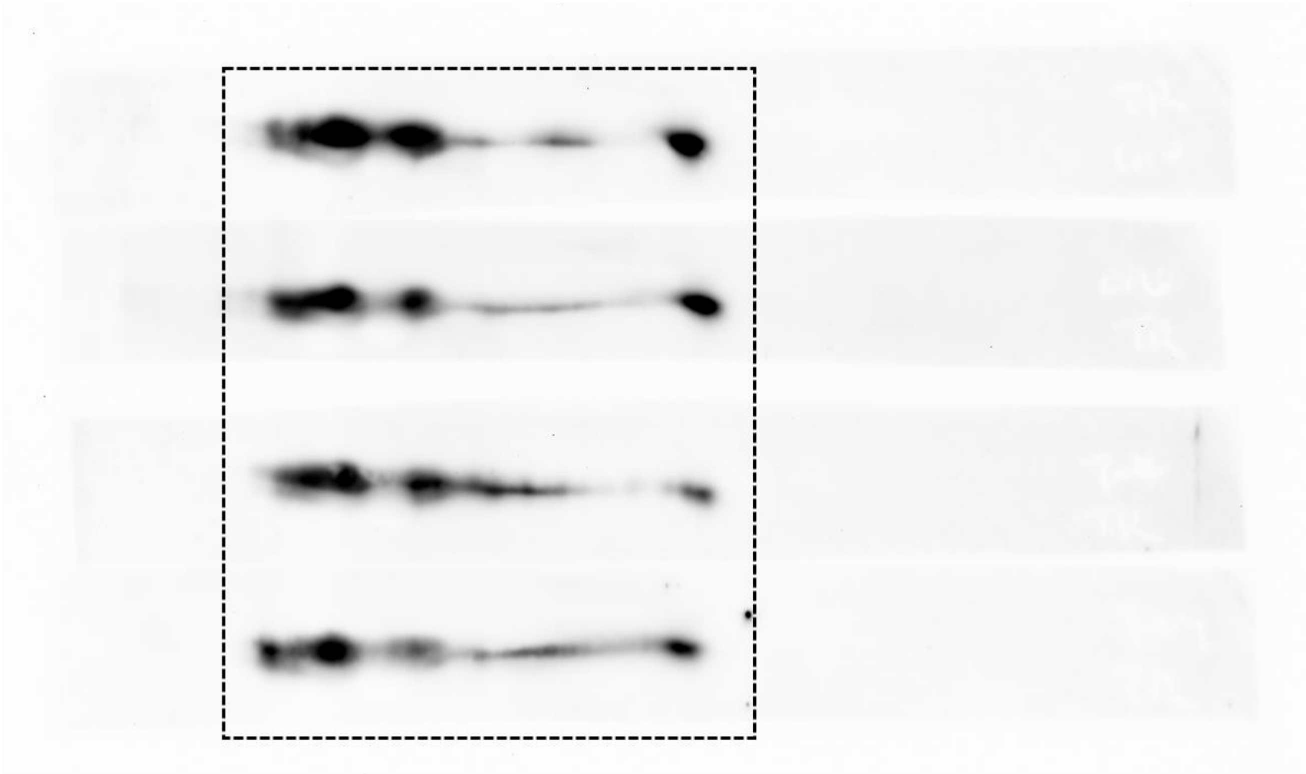

Original blots for the Figure 6B

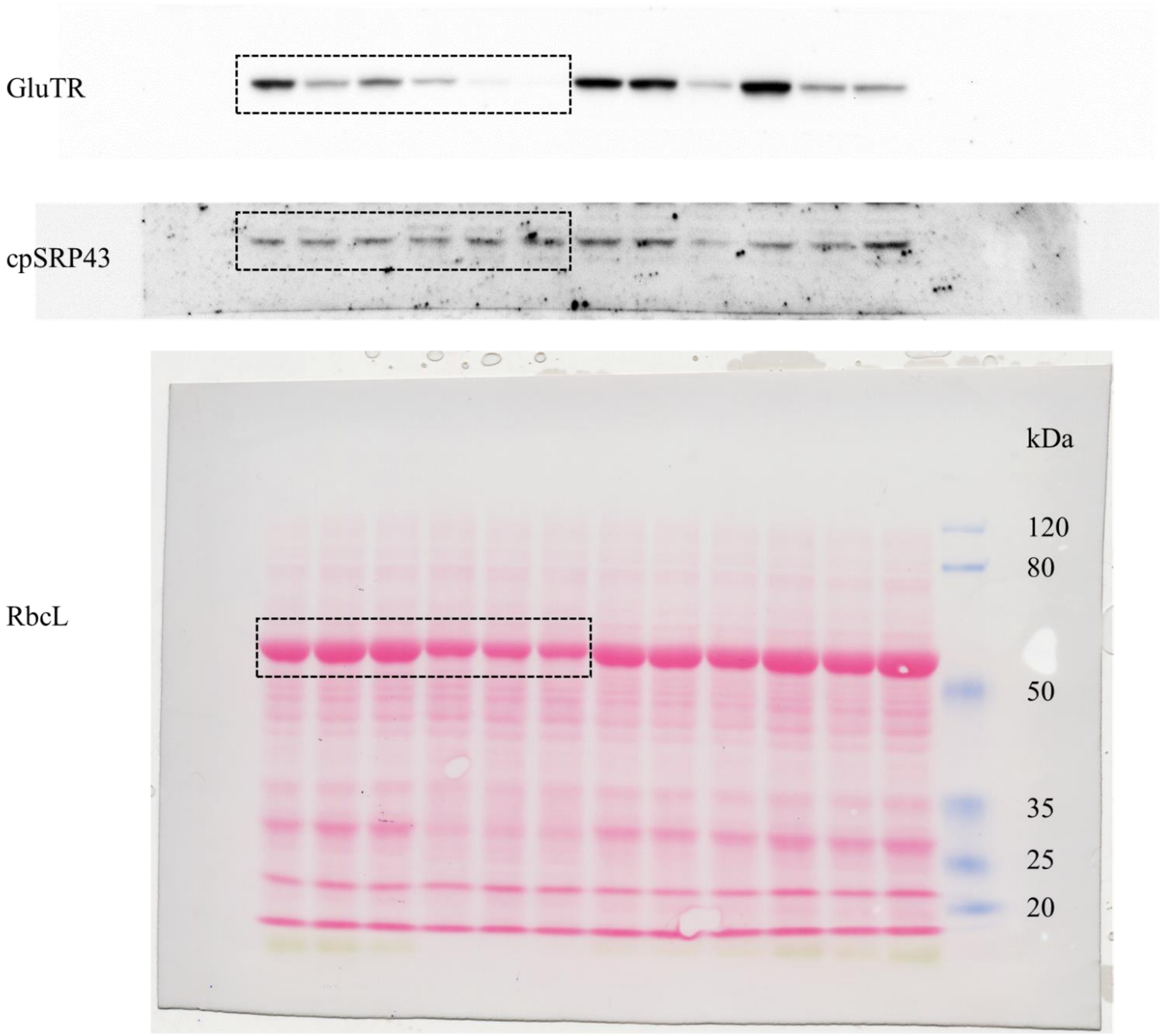

Original blots for the Figure 7A

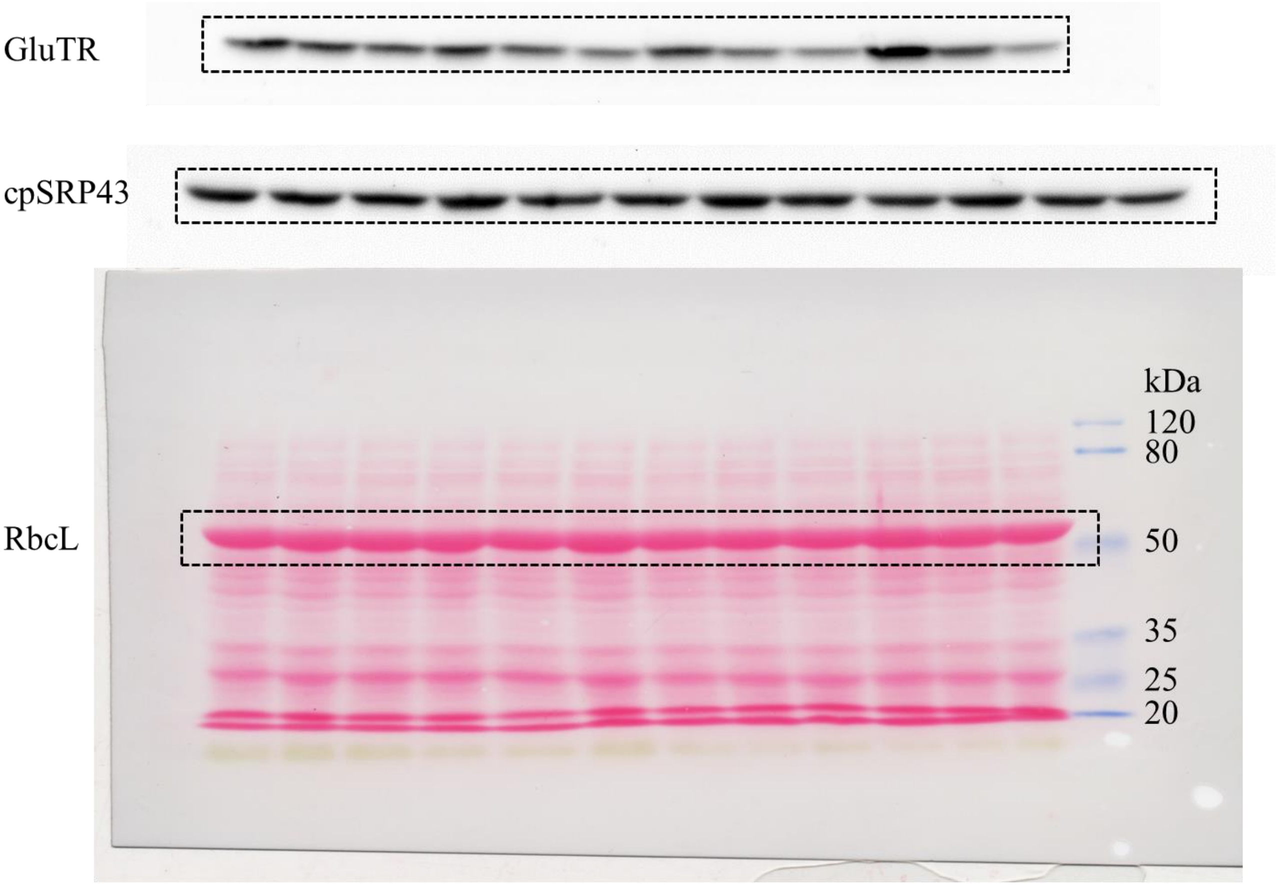

Original blots for the Figure 7A

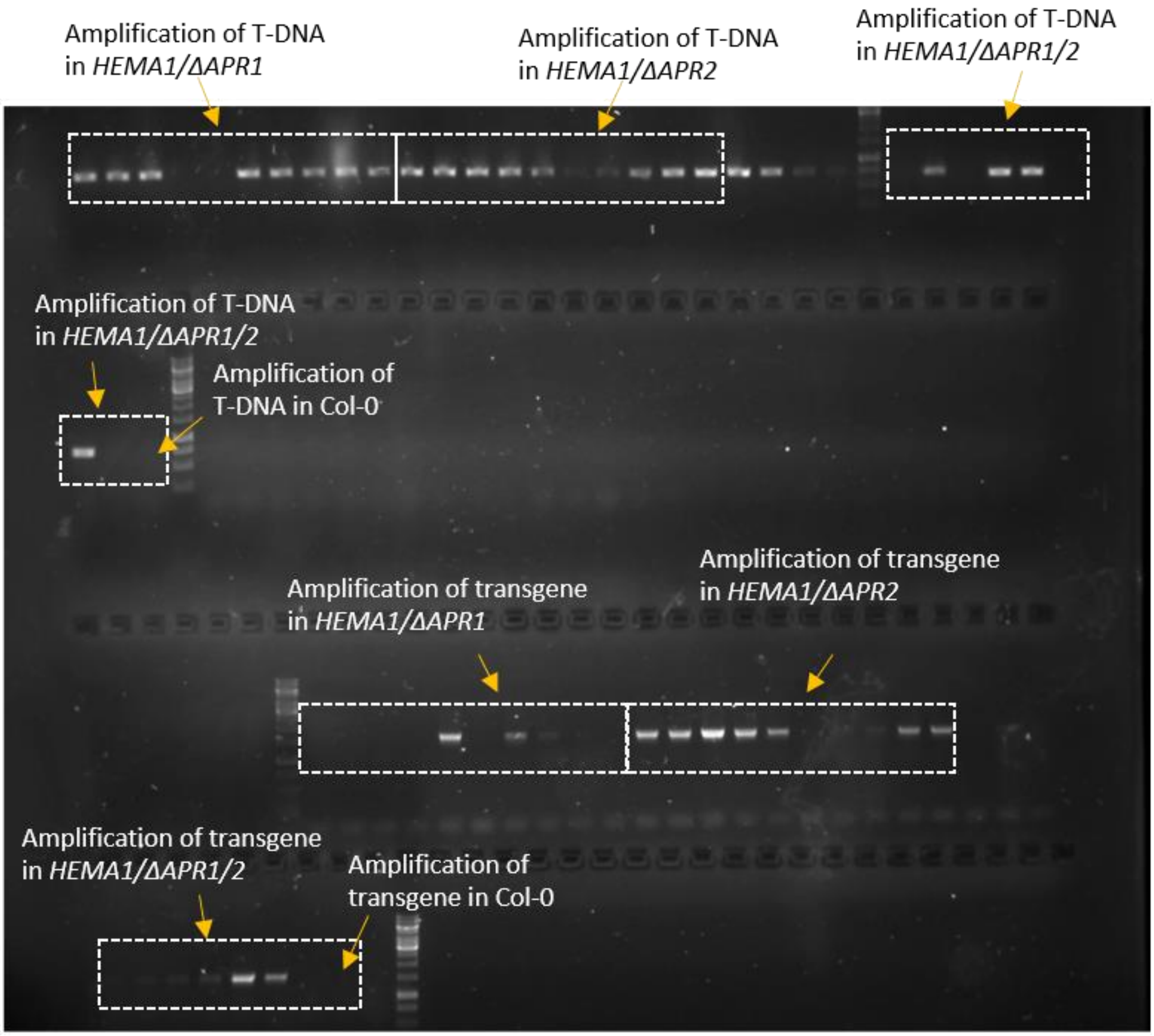

Original gels for the Figure S1

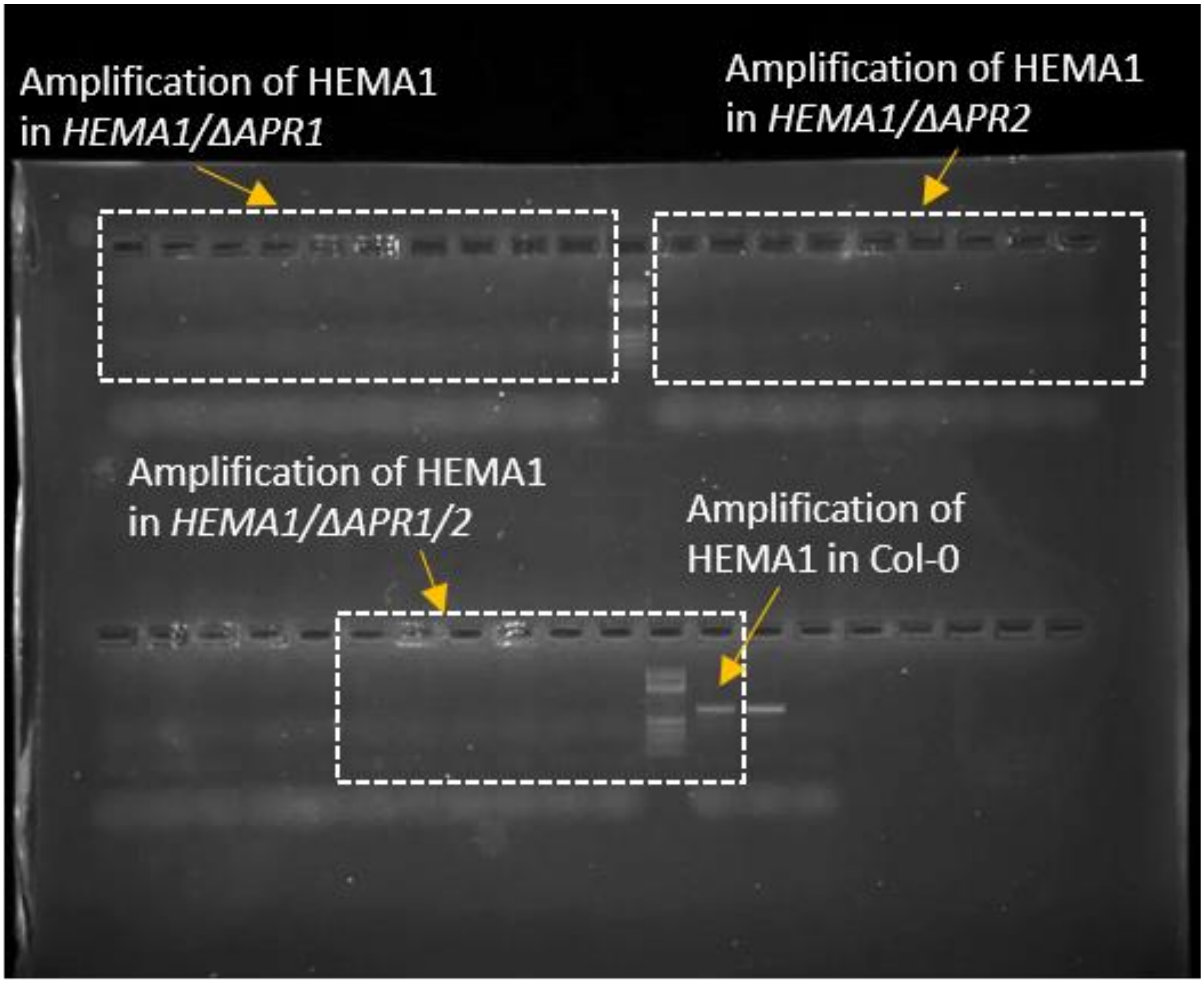

Original gels for the Figure S1

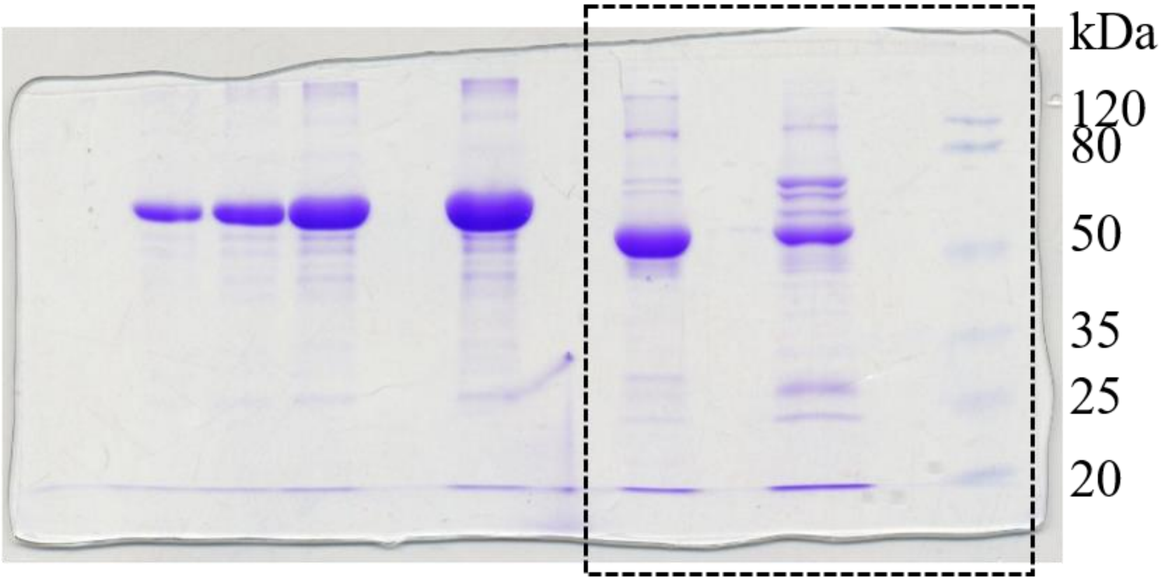

Original blots for the Figure S3
